# Personality traits and risky behavior among motorcyclists: An exploratory study

**DOI:** 10.1371/journal.pone.0225949

**Published:** 2019-12-05

**Authors:** Daniel Luiz Romero, Daniel Martins de Barros, Gabriel Okawa Belizario, Antonio de Pádua Serafim

**Affiliations:** 1 Department of Psychology, Methodist University of São Paulo, São Bernardo do Campo, Brazil; 2 Department and Institute of Psychiatry, University of São Paulo School of Medicine, São Paulo, Brazil; Universitat de Valencia, SPAIN

## Abstract

**Background:**

Personality traits have been associated with a series of dysfunctional behaviors, ranging from violence to drug abuse and other risky behaviors. However, few studies have investigated motorcycle riders’ personality traits, and no research using the psychobiological model of personality was found. Thus, we investigated the association between temperament and character traits and the occurrence of Motorcycle Accidents (MAs).

**Methods:**

This cross-sectional study was conducted with a randomly selected of 153 Brazilian motorcycle riders (116 male and 37 female) with a mean age of 31.8randomly selected from the driver’s license register, between 2015 and 2018. A sociodemographic questionnaire and the Temperament and Character Inventory (TCI) were used.

**Results:**

Of the 153 participants, 146 (95.4%) reported having been involved in previous accidents, with the main causes as follows: “other drivers’ careless behavior” (34.9%); “personal disrespect of traffic rules” (18.5%) and “personal careless behavior” (20.6%). Motorcyclists exhibited higher scores for the temperament factors of novelty seeking and persistence and lower scores for harm avoidance and reward dependence in comparison to the Brazilian population). Considering the reason for motorcycle use, the group of riders that used their motorcycles for work exhibited more temperament factors associated with risk behaviors than those who did not. It was observed that 68.9% of them had low Harm Avoidance (HA) factor scores, whereas 72.1% had high Novelty Seeking (NS) factor scores.

**Discussion:**

The present study highlighted the influences of different personality traits on behaviors, decision-making and risk attitudes that can be potentially harmful to an individual and others. The results provided evidence that a lack of knowledge and experience in riding a motorcycle or any other vehicle, combined with personality traits, contribute to the adoption of risky behaviors that may act as triggers for most causes of Motorcycle Accidents.

## Introduction

Over the last 100 years, there has been substantial progress in the development of technologies regarding the safety of automotive vehicles. This progress has led, for example, to an accident reduction of approximately 51.1% in several developed countries between 2000 and 2010 [1]. However, the literature also emphasizes that despite improvements in the road environment and vehicles that have achieved great safety gains, the number of accidents remains high, resulting in a substantial number of deaths and sequelae; automobile accidents have been mainly associated with driver behavior. In general, studies have shown that risky driving is an important factor for traffic accidents [2,3]. These findings have been demonstrated for both experienced and novice drivers [2,4,5].

Considering only the number of accidents involving motorcyclists, the rates are worrying since the number of deaths and serious injuries constitutes an epidemic [6]. Brown et al. [7] mention that although addressing Motorcycle Accidents (MAs) is a complex task, such research can have both great personal and public safety impacts. The increase in the number of MAs, the consequent morbidity and mortality, and the incidence of sequelae resulting from MAs have become a public health issue in many countries [8,9].

In developing countries, such as Brazil, motorcycles have become increasingly popular since they are an economically advantageous option, a work tool for many people and an alternative to other modes of transportation for daily commuting in large cities [10]. In addition, there has been a proportional increase in the number of MAs and the resulting mortality rate from these accidents. Data have shown that between 1996 and 2011, the death rate of motorcycle riders increased by 742.5% [10]. Data from the Ministry of Health in Brazil state that the total number of deaths of motorcycle riders increased from 4,292 in 2003 to 12,040 in 2013. MAs deaths amount to 28% of the 45,000 deaths from ground transportation accidents in the country every year.

There are two main approaches for addressing MAs: the first one includes the use of safety equipment and the determination of risk factors [11]. Regarding the second approach, the main factors associated with motorcycle accidents include age, the motorcyclist’s lack of attention, poor motorcycle maintenance, motorcyclist´s careless driving, and disrespect of traffic rules among others [11,12,13], as well as risky driving attitudes, which is a common phenomenon in various types of drivers and is associated with psychosocial factors and personality aspects [14].

In this scope of personality, the studies include a series of dysfunctional behaviors, ranging from violence [15,16,17,18], drug abuse [10,19] and risky behaviors, which may result in a variety of accidents, including MAs [20, 21].

Personality dimensions such as Novelty-Seeking have also been associated with risky driving behavior [22]. The search for novelty is one of the defining characteristics of a human sensation-seeking personality [23]. Thus, the search for novelty is a characteristic constituted by the search for new sensations and the willingness to take physical, social, legal and financial risks in favor of such experiences. Novelty serves as a signal that provides motivation to seek rewards. Individuals seeking new experiences exhibit overwhelming motivational force that leads to a lower ability to control their actions, such as compulsive drug use. Individuals characterized as novelty-seekers often have high impulsivity, exploratory excitability, extravagance, and are unorganized [23].

Lucidi and colleagues [24] studied drivers´ behaviors considering three different age groups (young: n = 435; adult: n = 412; old: n = 439) in association with personality aspects. The results showed that risk driving was associated with personality traits and anxiety in young and adult drivers, while the search for sensations was associated with risky driving only in young drivers. In another study, researchers verified alcohol consumption and personality factors as predictors of risk driving in offending drivers compared to non-offenders. The results showed that increased factors such as sensation seeking were associated with hostility while driving [25].

Wong, Chung and Huang [4] developed an explanatory model based on personality traits to explain risky driving behaviors. Using this model with 683 young motorcyclists aged 18 to 28 years, the results indicated three primary personality traits of young motorcyclists: searching for sensation, amiability and impatience. The authors observed that high levels of sensation and impatience were associated with high risk taking. However, the presence of kindness was associated with a more prudent attitude.

Another study by Von Below [26], analyzed a sample of subjects involved in motorcycle accidents in Germany, and identified seeking excitement, hostility, lack of normalization and low altruism in 23% of the interviewees. In India, researchers investigated the prevalence of personality traits in 250 motorcyclists with a history of accidents and 210 controls of age using the International Personality Disorder Examination (IPDE)-ICD 10 module screening questionnaire, which includes nine personality traits. The results revealed that 85.9% of those interviewed had impulsive personality traits, and 82.72% had histrionic traits [27].

In fact, personality aspects have been shown to be important variables associated with motorcycle accident risk. However, we did not identify in the literature studies using Cloninger’s psychobiological model to investigate motorcyclists with a history of accidents. In this study, we included the Temperament and Character Inventory (TCI) [28] in its Brazilian version [29], which is used to assess personality traits of the general population and patients with mental disorders. The TCI enables a comprehensive assessment of personality traits by measuring seven dimensions of personality (four temperament dimensions and three character dimensions) that are moderately heritable and associated with distinct brain networks and psychological characteristics [28].

The temperament dimensions represent stable, heritable, neurobiological dispositions for learning automatic behavioral reactions in response to specific environmental stimuli (danger, novelty, and reward); the dimensions include novelty seeking, harm avoidance, reward dependence and persistence. The character traits, i.e., self-directedness, cooperativeness and self-transcendence, change with age and are more closely associated with higher cognitive processes, including interpretation and formal construction [28]. This model is important because the dimensions are associated with a series of risk behaviors ranging from violence to accidents, with an emphasis on impulsiveness [15,30,31,32].

Until this moment, we have not identified studies that used TCI to investigate temperament and character traits as a risk factor for the occurrence of accidents with motorcyclists; thus, one goal of this study is to fill this gap in. In addition, investigating the relationship between personality traits and motorcyclist risky behavior can contribute to understanding the reality of these subjects, and to a better planning and decision-making aimed at reducing significant traffic accidents and their consequences.

Thus, we investigated the potential association between temperament and character traits according to the psychobiological theory of Cloninger et al. [28], using the Portuguese version of TCI [29] and the risk of motorcycle accidents in the metropolitan region of Sao Paulo, Brazil. We hypothesize that increased novelty seeking and diminished harm avoidance may be associated with increased motorcycle accidents.

## Methods

### Participants

This cross-sectional study was conducted with a convenience sample of 153 Brazilian motorcycle riders randomly selected from the driver’s license register, between 2015 and 2018. The participants were aged between 18 and 60 years old, both genders and had different levels of education (see Table 1).

**Table 1:** Sociodemographic data of 153 motorcycle riders.

Variable		MD (SD)	N	%
Age		31.8 (8.08)		
Gender				
	Male		116	75.8
	Female		37	24.2
Schooling				
	Some elementary school		4	2.6
	Elementary school		6	3.9
	Some high school education		9	5.9
	High school education		55	35.9
	Some higher education		33	21.6
	Higher education		35	22.9
	Some graduate studies		6	3.9
	Graduate studies		5	3.3
Marital Status				
	Single		74	48.4
	Married		73	47.7
	Separated/Widowed/Divorced		6	3.9
Motorcycle use				
	Work		61	39.9
	Daily transportation		116	75.8
	Leisure		88	57.5
	Km travelled per day	82,4 (72,3)		
Has had an accident?	Yes		146	95.4
	Number of accidents	4.71 (3.69)		
Reason for the accident				
	Motorcyclist’s lack of attention		13	8.9
	Other drivers’ careless behavior		51	34,9
	Poor motorcycle maintenance		4	2.7
	Personal disrespect of traffic rules		27	18.5
	Poor road conditions		6	4.1
	Use of alcohol and drugs		15	10.3
	Personal careless behavior		30	20.6
Consequences of accidents for health				
	Need for pins and plates		29	19
	Surgical interventions		31	20,3
	Light scalings		120	78,4
	Absence from work		55	35,9
	Comatose status		2	1,3
	Hospital admission		39	25,5
	Fractures		66	45,2
	Spinal injury		14	9.5

All participants signed informed consent forms for the purposes of voluntary research participation, and agreement for data to be used in this study respecting the participant´s confidentiality. The entire study protocol was approved by the Research Ethics Committee of Methodist University of São Paulo under protocol number 44750015.9.0000.5508 on 05/21/2015.

### Instrument*s*

#### Sociodemographic questionnaire

We used a questionnaire with sociodemographic variables such as gender, marital status, schooling, motorcycle use, accident occurrence and reason for the accident.

#### Personality traits

The Temperament and Character Inventory (TCI) is a 240-item self-report questionnaire that assesses four temperament dimensions and three character dimensions using a true/false model (number 1 was assigned for ‘true’ and 0 for ‘false’). The minimum and maximum scores are expressed in the description of each dimension. The final result is verified by the mean of the scores in each dimension. For this study we applied its Brazilian Portuguese version of the TCI [29] and compare the data with the control group results from a recent Brazilian study [15].

#### Temperament dimensions

NS—Novelty Seeking, scores from 0 to 40: is characterized by impulsive behavior, an exploratory search for novelty and pleasure and low tolerance for frustration. In these situations, these individuals may show an explosive attitude with frequent emotional disinhibition; HA—Harm Avoidance: score from 0 to 35: is characterized by anticipated pessimistic concerns with future events, fear of the unknown and shyness with strangers, which leads to avoidance behavior, whereby the individual avoids challenges or involvement in new situations; RD—Reward Dependence, score from 0 to 24: is characterized by fondness for social relationships and value for external approval; P—Persistence, score from 0 to 8: the hereditary tendency to persist in responding in a certain way, despite intermittent reinforcement (fatigue and/or frustration).

#### Character dimensions

SD—Self-directedness, score from 0 to 44: is defined as the individual capacity to adapt to different situations in order to obtain personal objectives. It also encompasses the potential of committing oneself to these objectives and taking responsibility for one's own actions; C—Cooperativeness, score from 0 to 42: is the capacity to understand and accept others, in contrast to self-centeredness and hostility; ST—Self-transcendence, score from 0 to 33: is associated with the presence of spirituality, that is, the feeling of belonging to a unified whole. This involves a state of "unified consciousness" in which everything is part of one totality, leading to acceptance, identification, or spiritual union with nature and its source.

Note: The motorcycle riders’ TCI results were compared to the normal range data for the Brazilian population [15]: range data expressed in Mean and Standard Deviation MD (SD): novelty seeking, 18.2(2.6); harm avoidance, 16.6(2.4); reward dependence, 16.2(1.2); persistence, 5.3(0.7); self-directedness, 35.5(1.4); cooperativeness, 34.2(1.5); and self-transcendence, 19.8(1.6) [15].

### Procedures

**Fig 1:**
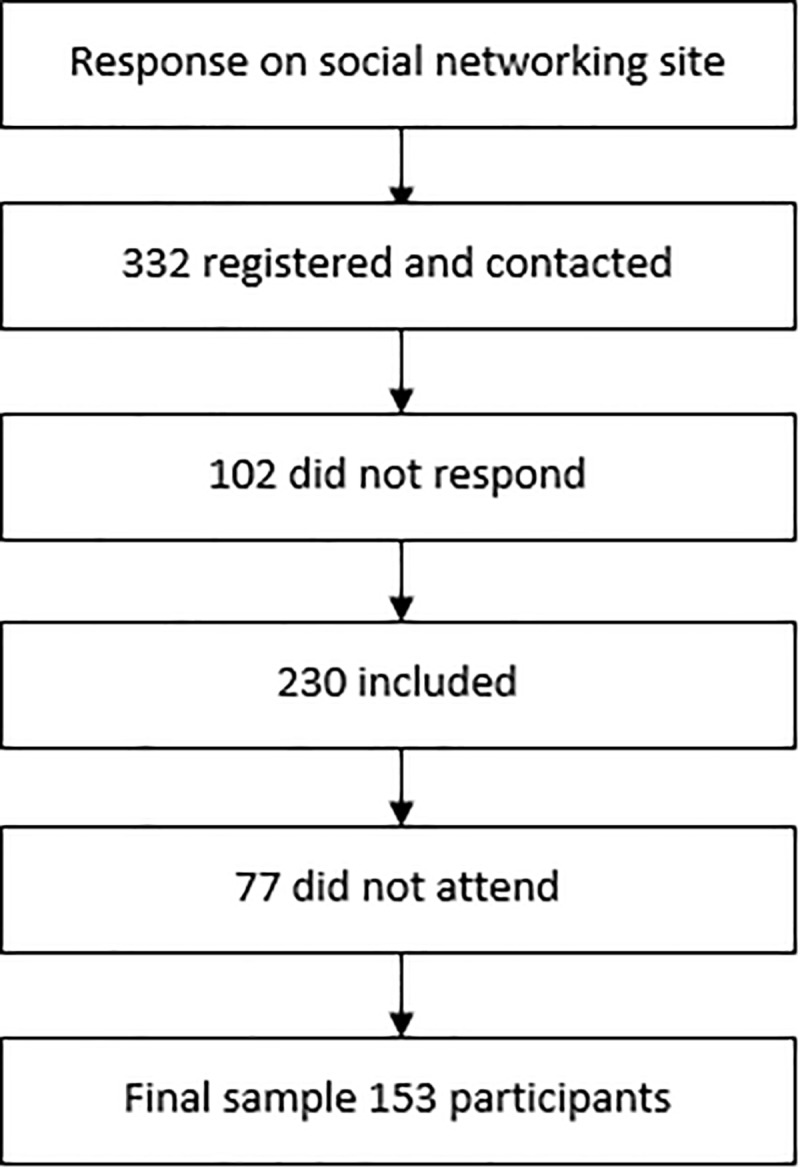
Flow diagram of the sample.

Initially, the participants responded to an electronic form through a social networking site regarding the use of motorcycles. After this stage, the researchers contacted 332 individuals via e-mail explaining the details of the study; 230 individuals responded. Then, the dates and locations for data collection (application of the instruments) were scheduled. Although 230 individuals were included, only 153 subjects actually attended the scheduled appointment (Fig 1).

### Statistical analysis

The statistical analysis was performed using SPSS for Windows version 25. The demographic data was presented in the form of percentage. For each variable of TCI, the minimum and maximum values, mean and standard deviation were calculated. To assess the normality of the data, we used the Kolmogorov-Smirnov test. In order to compare the mean between temperament and character scores of motorcyclists, and the Brazilian data, we used the Student’s *t*-test. To compare the low NS and high NS groups, and low HA and high HA groups by age, gender, reason for the accident and health consequences of the accident, we used a Mann-Whitney Test (Mean and standard deviation) and Pearson’s chi-square Test or Fisher’s exact test for Number of observations (percentage). The significance level adopted in this study was 0.05.

## Results

### Sociodemographic data

The Table 1 shows that, according to the studied sample, the main reason for the accidents was other careless drivers.

The Mean age and Standard Deviation was 31.8 (8.08), and most motorcyclists had finished their high school education and were male. Most of the participants used a motorcycle as their main means of transportation. Accidents had happened with 146 subjects (95.4%), with 74.6% of the men and 100% of the women. Regarding the causes of the accidents, the participants reported “other drivers’ careless behavior” (34.9%), “personal disrespect of traffic rules” (18.5%) and “personal careless behavior” (20.6%) as the most common causes. Regarding the severity of the accidents, 45.2% of the participants had fractures, 19% had to use pins and/or plates, 9.6% had spinal injuries and 20.3% needed surgery.

### Personality traits

The Table 2 shows the comparison between the TCI scores and the standardized data from the Brazilian population.

**Table 2:** Temperament and character factors.

Variable	Motorcyclists (n = 153) MD (SD)	Brazilian data MD (SD)	P-value*
Temperament			
NS	**20.3 (5.8)**	18.2 (2.6)	**<0.001**
HA	14.96 (5.4)	16.6 (2.4)	0.006
RD	**12.76 (3.2)**	16.2 (1.2)	**< 0.001**
P	5.8 (1.6)	5.3 (0.7)	0.002
Character			
SD	**28.9 (6.8)**	35.5 (1.4)	**< 0.001**
CO	**29.1 (6.1)**	34.2 (1.5)	**< 0.001**
ST	18.2 (5.6)	19.8 (1.6)	0.007

NS (novelty seeking), HA (harm avoidance), RD (reward dependence), P (persistence), SD (self-directedness), CO (cooperativeness) and ST (self-transcendence).

*p Student’s t-test

These results demonstrated that the group of motorcycle riders in general has a tendency to explore and make impulsive decisions, often adopting more risk behaviors as the NS scores increase (p<0.001 Student’s *t*-test). In addition, they appear to be less concerned about their actions and substantially more optimistic in comparison to the Brazilian population.

As there were lower scores for HA and higher scores for NS, we conducted a covariate analysis of these personality traits by age, gender, reason for the accident and health consequence of the accident to detect possible influences of these variables (Tables 3 and 4).

**Table 3:** Comparison of the low NS and high NS groups by age, gender, reason for the accident and health consequences of the accident.

Variable		Low NS	High NS	P-value^1,2^
Age*		34.29 (8.90)	30.42 (7.25)	0.008 ^A^
Gender**	*Female*	11 (19.6)	26 (26.9)	0.319 ^1^
	*Male*	71 (73.2%)	45 (80.4%)	
Motorcyclist’s lack of attention	No	37 (40.7)	54 (59.3)	0.207 ^1^
	Yes	19 (30.6)	43 (69.4)	
Other drivers’ careless behavior	No	17 (38.6)	27 (61.4)	0.740 ^1^
	Yes	39 (35.8)	70 (64.2)	
Poor motorcycle maintenance	No	54 (36.5)	94 (63.2)	1.000 ^2^
	Yes	02 (40)	03 (60)	
Personal disrespect of traffic rules	No	51 (45.9)	60 (54.1)	**> 0.001** ^**2**^
	Yes	05 (11.9)	**37 (88.1)**	
Poor road conditions	No	34 (39.5)	52 (60.5)	0.393 ^1^
	Yes	22 (32.8)	45 (67.2)	
Use of alcohol and drugs	No	55 (40.1)	82 (59.9)	**0.006** ^**2**^
	Yes	01 (6.3)	**15 (93.8)**	
Personal careless behavior	No	49 (50)	49 (50)	**> 0.001** ^**1**^
	Yes	07 (12.7)	**48 (87.3)**	
Speeding	No	52 (46)	61 (54)	**> 0.001** ^**2**^
	Yes	4 (10)	**36 (90)**	
Need for pins and splints	No	50 (43.3)	74 (59.7)	**0.048** ^**1**^
	Yes	06 (20.7)	**23 (79.3)**	
Surgical procedures	No	51 (41.8)	71 (58.2)	**0.008** ^**1**^
	Yes	05 (16.1)	**26 (83.9)**	
Mild bruises	No	12 (36.4)	21 (63.6)	0.974 ^1^
	Yes	44 (36.7)	76 (63.3)	
Absence from work	No	40 (40.8)	58 (59.2)	0.149 ^2^
	Yes	16 (29.1)	39 (70.9)	
Comatose status	No	56 (37.1)	95 (62.9)	0.533 ^2^
	Yes	00 (00)	02 (100)	
Hospital admission	No	48 (42.1)	66 (57.9)	**0.016** ^**1**^
	Yes	08 (20.5)	**31 (79.5)**	
Fracture	No	43 (49.4)	44 (50.6)	**> 0.001** ^**1**^
	Yes	13 (19.7)	**53 (80.3)**	
Spinal injury	No	54 (36)	96 (64)	0.555 ^2^
	Yes	02 (66.7)	01 (33.3)	

* Mean (standard deviation)

** Number of observations (percentage)

^A^Mann-Whitney Test; NS (novelty seeking).

*p*^1^ = Pearson’s chi-square Test

*p*^2^ = Fisher's exact test

**Table 4:** Comparison of the low HA and high HA groups by age, gender, reason for the accidents and health consequences of the accident.

Variable		Low HA	High HA	P-value
Age*		31,45(8.11)	32.35 (8.07)	0.323 ^**A**^

Gender**	*Female*	**28 (75.7)**	09 (24.3)	**0.010** ^**1**^
	*Male*	60 (51.7)	56 (48.3)	
Motorcyclist’s lack of attention	No	48 (52.7)	43 (47.3)	0.148 ^1^
	Yes	40 (64.5)	22 (35.5)	
Other drivers’ careless behaviour	No	24 (54.5)	20 (45.5)	0.637 ^1^
	Yes	64 (58.7)	45 (41.3)	
Poor motorcycle maintenance	No	83 (56.1)	65 (43.9)	1.000 ^2^
	Yes	05 (100)	00 (00)	
Personal disrespect of traffic rules	No	54 (48.6)	57 (51.4)	**> 0.001** ^**1**^
	Yes	**34 (81)**	65 (19)	
Poor road conditions	No	45 (52.3)	41 (47.7)	0.141 ^1^
	Yes	43 (64.2)	24 (35.8)	
Use of alcohol and drugs	No	74 (54)	63 (46)	**0.010** ^**1**^
	Yes	**14 (87.5)**	02 (42.5)	
Personal careless behaviour	No	46 (46.9)	52 (53.1)	**> 0.001** ^**1**^
	Yes	**42 (76.4)**	13 (23.6)	
Speeding	No	57 (50.4)	56 (49.6)	**0.003** ^**1**^
	Yes	**31 (77.5)**	09 (22.5)	
Need for pins and splints	No	66 (53.2)	58 (46.8)	**0.026** ^**1**^
	Yes	**22 (75.9)**	07 (24.1)	
Surgical procedures	No	65 (53.3)	57 (46.7)	**0.035** ^**1**^
	Yes	**23 (74.2)**	08 (25.8)	
Mild bruises	No	16 (48.5)	17 (51.5)	0.236 ^1^
	Yes	72 (60)	48 (40)	
Absence from work	No	54 (55.1)	44 (44.9)	0.420 ^1^
	Yes	34 (61.8)	21 (38.2)	
Comatose status	No	86 (57)	65 (43)	0.508 ^2^
	Yes	02 (100)	00 (00)	
Hospital admission	No	58 (509)	56 (49.1)	**0.005** ^**1**^
	Yes	**30 (76.9)**	09 (23.1)	
Fracture	No	41 (47.1)	46 (52.9)	**0.003** ^**1**^
	Yes	**47 (71.2)**	19 (28.8)	
Spinal injury	No	87 (58)	63 (42)	0.575 ^2^
	Yes	01 (33.3)	02 (66.7)	

* Mean (standard deviation)

** Number of observations (percentage)

^**A**^Mann-Whitney Test

HA (harm avoidance).

*p*^1^ = Pearson’s chi-square Test

*p*^2^ = Fisher's exact test

The results showed statistically significant differences: the group of motorcycle riders who had higher NS scores was younger, and women had lower HA scores than men (p = 0.008, Mann-Whitney Test). Concerning the reasons for the accidents, the combination of high NS and low HA scores was associated with behaviors such as personal careless behavior (p<0.001, Pearson’s chi-square Test), high speed driving and disrespect of traffic rules (p<0.001, Fisher's exact test) and the use of alcohol and drugs (p = 0.006, Fisher's exact test). Moreover, it was associated with worse health consequences, such as hospital admission, surgical intervention, fractures and the need for pins or splints to be implanted in bones.

Considering the reason for motorcycle use, as shown in Table 5, the results demonstrated that the group of riders that used their motorcycles for work exhibited more temperament factors associated with risk behaviors than those who did not. It was observed that 68.9% of them had low HA factor scores, whereas 72.1% had high NS factor scores (p = 0.021, Pearson’s chi-square test).

**Table 5:** Comparison of the low NS, high NS, low HA and high HA groups by motorcycle use.

Variable		Low NS	High NS	p	Low HA	High HA	P-value^1^
Work **	No	39 (42.2)	53 (57.6)	0.068 ^1^	46 (50)	46 (50)	**0.021** ^**1**^
	Yes	17 (27.9)	44 (72.1)		**42 (68.9)**	19 (31.1)	
Daily transportation**	No	15 (40.5)	22 (59.5)	0.568 ^1^	25 (67.6)	12 (32.4)	0.155 ^1^
	Yes	41 (35.3)	75 (64.7)		63 (54.3)	53 (45.7)	
Leisure**	No	24 (36.9)	41 (63.1)	0.943 ^1^	34 (52.3)	31 (47.7)	0.263 ^1^
	Yes	32 (36.4)	56 (63.6)		54 (61.4)	34 (38.6)	

** Number of observations (percentage)

*p*^1^ Pearson’s chi-square test

NS (novelty seeking)

HA (harm avoidance)

## Discussion

In general, it is agreed in the literature that risk behaviors in various social contexts also impact health risks. Studies have shown that personality traits and attitudes towards traffic safety predict dangerous driving behaviors and involvement in accidents [3,7,14,24, 25, 30]. However, this association has not been well investigated among motorcyclists using the psychobiological model of temperament and character. Thus, the main purpose of this study was to investigate the association between personality traits according to the psychobiological model (temperament and character) proposed by Cloninger et al. [28] and the risk of motorcycle accidents.

Concerning sociodemographic data, our sample of motorcycle riders is similar to those reported in the literature regarding gender, with a clear male prevalence [11,33, 34], and regarding age, with a mean of 31 years old, representing a young, productive population group. According to Reichenheim et al. [31] deaths related to motorcycle accidents are more common among young adults aged 20 to 39 years. Santos et al. [35] reported a higher prevalence in the age range of 25 to 34 years, and a study conducted by Oliveira and Souza [34] showed a higher prevalence in the age range of 20 to 29 years.

An unexpected finding in this study was the high number of accidents in our sample, with almost all participants reporting having been in an accident before. The rate reported in the present study is very different from those of other countries. In the European Community´s Annual Accident Report [36] the accident rate is 2.3% and in Canada, rates are approximately 10% [6]. Brazil has the fifth highest rate of traffic accidents in the world, and although motorcycles represent 27% of the national fleet of motor vehicles in Brazil, according to the annual report of the Damage Insurance Personal Injuries Caused by Land Vehicle Vehicles–DPVAT [37], they account for the highest number of accidents and victims. Between January and December 2017, there were a total of 383,993 MAs, with 41,151 fatalities [37]. Our data corroborate the information provided by the World Health Organization (WHO) [38], which showed that in 2013, more than 286,000 motorcyclists were killed worldwide by traffic accidents.

Concerning the reasons for the accidents, the results are similar to those of Pordeus et al. [39]. In our studied sample, “motorcyclist’s lack of attention” was the reason associated with almost half of the accidents, whereas in the compared study, the same reason may be responsible for approximately one third of the accidents. When comparing speeding as a reason, there is inconsistency among the data, as in Pordeus et al. [39], speeding reason was responsible for almost half of the accidents, whereas in our sample, speeding was the reason for only one fourth of the accidents. These results are similar to those found by Silva et al. [40] in their study in the cities of Londrina and Maringa, Brazil, in which approximately one fourth of the accidents were caused by speeding.

Another important finding concerns the individual who held responsibility for an accident, which involves the assumption that he or she performed the behavior that led to the accident. In the current study, much more than half of the sample reported that the cause of the accident was the other drivers’ careless behavior, whereas one third of the interviewed subjects reported that they were responsible for the accident, as they personally had been careless. These data are in agreement with a study developed by the Brazilian Association of Manufacturers of Motorcycles, Motorized Bikes, Scooters, Bikes and Others—ABRACICLO [41], which reviewed inspection data and concluded that more than half of the accidents were caused by the careless behavior of other drivers.

As such, we can argue whether the personality traits of the studied motorcycle riders, plus other drivers’ carelessness, may be the main risk factors related to the causes of accidents.

As noted in this study, the group of motorcycle riders presented differences in all temperament and character traits compared to the general population. People who show a high level of NS are more prone to adopt risk behaviors, especially concerning regulation and adaptation of actions and impulses [15,42]. In the case of the studied population, such data are related to more dangerous driving. These findings corroborate the study conducted by Ulleberg and Rundmo [43], which pointed to the influence of personality traits on the behaviors that indirectly form an attitude that promotes riskier driving. Studying a group of motorcycle riders with a questionnaire that assessed the traits of impulsively seeking, violence and risk behaviors, the authors identified that riders involved in accidents presented higher scores in these traits [44].

Although focusing the study on personality diagnosis, authors et al identified impulsivity as a common trait in motorcycle accident cases [45]. Another study showed that high scores for sensations and manifestations of anger were more associated with risky driving and accidents, and sensation seeking, aggression, and impulsivity [2 Iversen, 2004]. According to Svrakic et al. [46], these characteristics were responsible for adopting riskier behaviour owing to impulsiveness, carelessness, extreme confidence and a lack of response to dangerous situations. In addition, drivers showed unrealistic optimism when exposed to the possibly of severe consequences from a dangerous situation. This relationship results from the combination of a high level of novelty seeking and a low level of harm avoidance, as observed in our own results.

As novelty seeking increases, motorcycle riders present greater vulnerability to situations and may manifest episodes of anger and take impulsive attitudes when faced by possible punishment. Regarding lower harm avoidance, it was observed that riders are more optimistic, less concerned and show greater confidence when put in situations of eminent danger. These results reinforce the foundations of the literature regarding the association of personality traits and risk behavior since temperament refers to automatic emotional responses or individual differences in the strength of the impulses underlying basic emotions that are moderately stable over time. The adventurer temperament profile, with a high demand for novelty and low damage prevention, is known to be predisposed not only to adventure but also to risk behaviors [28].

Character factors are the traits resulting from experiences learned through social interactions [28]. It was observed that the motorcycle riders presented significantly lower scores for the character factors. The group of motorcycle riders presented difficulties in regulating and adapting their behaviours, low tolerance, a reduced capacity to accept different behaviours and opinions, over-criticism and difficulty reflecting [28,46,47].

These data confirmed the findings from previous studies that have also identified the specific characteristics of personality traits of the studied groups, such as excitement seeking, hostility and low altruism, and associations with antisocial and borderline personality disorders, obsessive compulsive and paranoid disorders [12,26,47,48].

Another important finding was the low cooperativeness score among the motorcyclists. Low scores on this trait suggest difficulty in accepting oppositional opinions and behaviors, little tolerance and empathy, insensibility, and over-criticism and are associated with personality disorders. The literature emphasizes that the interactions between character traits are related to personality disorders, and low scores in cooperativeness and self-directedness, for example, are described as traits present in all clusters of personality disorders [46].

An important additional observation in this study was the combination of high NS and low HA scores in association with low self-directedness scores, which seems to maximize the tendency to blame others. Thus, the occurrence of accidents seems to be strongly influenced by the tendency to blame others, including personality traits such as irrationality, little concern for consequences, irresponsibility, unrealistic behavior low emotional distress, and impulsiveness [14,47,49]. Conversely, more prudent people normally take responsibility for an action or infraction, assuming a more rational position, analyzing the situation, recognizing their errors, coping with the distress generated by them, adopting realistic behavior and adapting to societal standards [47].

### Limitations

The limitations of the study were not having a control group without a history of accidents and having a less robust sample than would be desired. Also, using self-report methods aren´t as reliable as using official records, and therefore is a significant limitation of the present study. Another limitation of the study is the lack of corrections for multiple comparisons, mainly due to our small sample size, which could lead to some false positive results, although these variables are not necessarily related to each other and correcting could generate false negatives.

Even so, considering the high prevalence of motorcycle accidents all over the world, we do think this sample was representative and the results from the study can be extrapolated to the motorcycle population in general. There are also very few studies involving the personality traits of drivers of other motor vehicles; therefore, additional studies would certainly provide interesting insights.

### Conclusion

The present study highlighted the influences of different personality traits on behaviors, decision-making and risky attitudes that can be potentially harmful to an individual or others. It was demonstrated that a lack of knowledge and experience in riding a motorcycle or any other vehicle, combined with personality traits, contribute to the adoption of risky behaviors that may act as triggers for most causes of motorcycle accidents. The findings in this study provide relevant information for public health, especially for those who ride a motorcycle as part of their jobs.

## Supporting information

S1 Table Personality and motorcyclists database. (PDF)
