# Supplementary material for: Personality traits and risky behavior among motorcyclists: An exploratory study
Source: PLoS One. 2019 Dec 5;14(12):e0225949. doi: 10.1371/journal.pone.0225949 (PMC6894775; doi:10.1371/journal.pone.0225949)
Supplement: S1 Table — (PDF) [file pone.0225949.s001.pdf]

Nome	Sexo	Idade	Escolaridac	Est.Civil	Filhos	Nºfilhos	Conhece_c	Fez_curso_
S - 1	M	32	6	2	2	2	2	2
S - 10	M	36	4	1	1	0	1	1
S - 100	M	18	4	1	1	0	2	1
S - 101	M	35	4	1	2	1	2	2
S - 102	F	26	5	1	2	1	2	1
S - 103	M	31	8	2	2	1	2	2
S - 104	M	28	2	1	2	1	2	1
S - 105	M	28	4	2	2	2	2	1
S - 106	M	23	5	1	2	2	2	2
S - 107	M	28	6	1	1	2	2	2
S - 108	F	28	6	1	1	0	2	1
S - 109	M	40	6	2	2	2	2	2
S - 11	M	38	4	1	1	0	2	1
S - 110	M	24	4	2	2	1	2	1
S - 111	F	26	5	1	1	0	1	1
S - 112	F	27	4	1	2	1	2	1
S - 113	F	25	6	1	1	0	2	1
S - 114	F	27	6	1	1	0	2	1
S - 115	F	31	5	2	1	0	1	1
S - 116	F	23	4	1	1	0	2	1
S - 117	F	27	5	2	1	0	2	1
S - 118	F	35	5	1	1	0	2	2
S - 119	M	27	4	1	1	0	2	1
S - 12	M	32	6	2	2	1	2	1
S - 120	F	33	6	2	2	2	2	2
S - 121	F	38	8	1	1	0	2	2
S - 122	F	46	7	2	2	3	2	1
S - 123	F	31	5	1	1	0	2	1
S - 124	M	34	8	2	1	0	2	2
S - 125	F	40	4	3	2	3	2	2
S - 126	M	26	4	2	1	0	2	2
S - 127	F	35	7	2	2	1	2	2
S - 128	F	28	6	1	1	0	2	1
S - 129	M	21	5	1	1	0	1	1
S - 13	M	42	6	2	2	2	2	1
S - 130	M	30	4	1	1	0	2	1
S - 131	M	26	5	1	2	1	2	1
S - 132	M	27	5	1	1	0	2	1
S - 133	F	32	6	2	1	0	2	1
S - 134	F	27	7	1	1	0	2	1
S - 135	F	28	4	2	1	0	2	1
S - 136	F	25	5	1	1	0	1	1
S - 137	M	25	4	2	1	0	1	2
S - 138	F	29	5	1	1	0	2	1
S - 139	M	30	4	2	1	0	2	2
S - 14	M	24	3	2	2	1	2	2
S - 140	F	23	4	1	1	0	1	1
S - 141	M	34	6	2	1	0	2	2
S - 142	M	37	6	2	2	3	2	1

S - 143	M	42	6	2	2	1	2	2
S - 144	F	27	5	1	1	0	1	1
S - 145	F	30	6	2	2	1	2	2
S - 146	M	25	5	1	1	0	2	1
S - 147	M	23	5	1	1	0	2	2
S - 148	M	32	6	1	1	0	2	2
S - 149	M	34	6	2	1	0	2	2
S - 15	M	56	6	2	2	2	2	2
S - 150	F	25	5	1	1	0	1	1
S - 151	M	39	6	3	2	1	2	1
S - 152	F	26	4	1	1	0	2	2
S - 153	M	41	8	2	2	2	2	2
S - 16	M	53	5	2	2	3	2	2
S - 17	M	28	4	1	1	0	1	1
S - 18	M	35	2	2	1	0	2	1
S - 19	M	31	4	1	2	2	1	1
S - 2	M	31	6	1	1	0	2	1
S - 20	M	40	3	2	2	2	2	2
S - 21	M	28	4	1	1	0	2	1
S - 22	M	36	4	1	1	0	2	2
S - 23	M	25	4	2	2	1	2	2
S - 24	M	48	1	2	2	2	2	1
S - 25	M	31	6	2	1	0	2	1
S - 26	M	31	4	2	2	1	2	2
S - 27	M	25	5	1	1	0	2	1
S - 28	M	31	4	2	1	0	2	1
S - 29	M	30	4	2	2	1	2	1
S - 3	M	45	6	2	2	1	2	1
S - 30	M	25	4	1	2	1	2	1
S - 31	M	23	4	1	1	0	2	1
S - 32	M	29	5	1	1	0	2	2
S - 33	M	29	3	2	2	3	2	1
S - 34	M	37	1	2	2	1	2	2
S - 35	M	33	3	1	1	0	2	1
S - 36	M	22	4	2	1	0	2	1
S - 37	M	48	4	3	1	0	2	1
S - 38	M	28	6	1	1	0	2	1
S - 39	M	25	5	1	1	0	2	2
S - 4	M	40	2	1	1	0	2	1
S - 40	M	30	4	1	1	0	2	1
S - 41	M	25	4	2	2	2	2	2
S - 42	M	53	2	2	2	1	2	1
S - 43	M	25	6	1	1	0	2	1
S - 44	M	24	4	1	1	0	2	2
S - 45	M	49	2	2	2	2	2	1
S - 46	M	34	5	2	2	2	2	1
S - 47	M	29	5	2	2	1	2	1
S - 48	M	23	5	1	1	0	2	1
S - 49	M	31	4	1	1	0	2	2
S - 5	M	25	5	1	1	0	1	1

S - 50	M	23	4	1	1	0	2	1
S - 51	M	33	4	2	2	2	2	1
S - 52	M	23	5	1	1	0	2	2
S - 53	M	22	5	1	1	0	2	1
S - 54	M	28	6	2	2	2	2	1
S - 55	M	29	4	2	2	1	2	2
S - 56	M	32	6	1	1	0	2	1
S - 57	M	31	4	2	2	1	1	1
S - 58	M	26	5	2	1	0	2	1
S - 59	M	28	3	1	2	4	1	1
S - 6	M	33	5	2	1	0	2	1
S - 60	M	22	5	1	2	1	2	1
S - 61	M	38	1	2	1	0	2	2
S - 62	M	29	4	1	1	0	2	1
S - 63	M	21	3	1	1	0	1	1
S - 64	M	33	4	1	2	1	2	2
S - 65	M	37	4	2	2	2	2	2
S - 66	M	34	5	2	2	3	2	1
S - 67	M	52	8	2	2	2	2	2
S - 68	M	48	7	3	2	2	2	1
S - 69	F	49	7	2	2	2	2	2
S - 7	M	36	4	2	2	2	2	1
S - 70	F	27	7	2	1	0	2	2
S - 71	F	53	6	2	2	1	2	2
S - 72	M	40	4	2	2	1	2	2
S - 73	M	39	4	1	1	0	2	2
S - 74	F	29	4	1	1	0	1	1
S - 75	F	20	4	1	1	2	1	2
S - 76	M	48	4	2	2	2	1	1
S - 77	M	29	5	1	1	0	2	2
S - 78	M	29	4	2	1	0	2	2
S - 79	M	37	4	3	2	2	2	2
S - 8	M	56	4	3	2	1	2	1
S - 80	M	38	4	2	1	0	2	2
S - 81	M	40	3	2	2	5	1	1
S - 82	M	26	3	2	1	0	2	2
S - 83	M	19	4	1	2	1	2	1
S - 84	M	36	3	2	2	5	2	2
S - 85	M	42	4	1	1	0	2	2
S - 86	M	26	5	1	1	0	2	1
S - 87	M	38	4	2	2	1	2	2
S - 88	M	30	4	2	2	1	2	1
S - 89	M	27	2	1	1	0	2	1
S - 9	M	25	5	1	1	0	2	1
S - 90	M	32	6	1	1	0	2	1
S - 91	M	43	6	2	2	1	1	2
S - 92	F	29	6	2	2	1	2	1
S - 93	M	32	6	1	1	0	2	1
S - 94	M	44	6	2	2	2	1	1
S - 95	F	29	6	2	2	1	2	1

S - 96	F	27	6	2	2	1	2	1
S - 97	F	25	6	1	2	1	1	1
S - 98	F	26	4	1	1	0	2	1
S - 99	M	22	1	2	2	1	2	1

Profissiona	Diario	Lazer	CNH	Pratica	KM_por_di	N.Acidente	sqrt_N.Acid	Falta_atenç
1	2	0	10	10	300	12	3,46	0
0	2	3	16	20	5	4	2,00	0
0	2	3	1	4	50	0	0,00	0
1	0	0	10	20	200	15	3,87	0
0	2	0	5	7	20	4	2,00	0
0	2	3	13	13	70	4	2,00	1
0	0	3	10	15	30	1	1,00	1
1	0	0	10	12	60	3	1,73	0
0	2	0	1	3	40	10	3,16	1
0	2	3	10	10	133	5	2,24	1
0	2	3	7	7	100	1	1,00	0
0	2	3	22	26	250	2	1,41	0
0	2	3	15	23	30	5	2,24	1
1	0	3	5	9	180	8	2,83	0
0	2	3	4	6	75	10	3,16	1
1	0	3	6	11	190	6	2,45	1
0	2	3	6	9	60	4	2,00	0
0	2	3	6	12	90	6	2,45	0
1	0	3	10	14	120	7	2,65	0
0	2	3	4	8	50	2	1,41	1
0	2	3	5	10	76	5	2,24	0
0	2	3	7	12	30	7	2,65	0
1	2	0	6	10	170	8	2,83	1
0	2	3	10	10	6	4	2,00	1
0	0	3	6	8	40	2	1,41	0
1	2	3	20	20	20	3	1,73	1
0	0	3	27	27	6	1	1,00	0
0	0	3	12	12	20	4	2,00	0
0	2	3	16	20	80	2	1,41	0
1	2	3	22	24	75	2	1,41	0
1	0	0	6	6	80	15	3,87	0
0	2	0	17	20	50	11	3,32	1
0	2	3	9	11	82	6	2,45	1
1	2	0	3	7	50	6	2,45	1
0	2	3	9	9	25	3	1,73	0
1	0	0	10	14	170	9	3,00	1
1	2	3	7	11	60	6	2,45	1
1	2	0	6	7	75	4	2,00	1
0	2	3	7	8	70	6	2,45	1
1	2	3	5	7	80	5	2,24	1
1	2	3	9	12	150	8	2,83	1
0	2	3	5	7	65	5	2,24	0
1	2	3	5	8	170	8	2,83	1
1	2	3	10	12	160	8	2,83	0
1	2	3	11	14	100	9	3,00	1
1	0	0	6	8	180	9	3,00	1
0	2	3	4	5	50	3	1,73	1
0	2	0	15	15	20	2	1,41	1
0	2	0	19	21	7	0	0,00	0

0	2	3	10	10	35	1	1,00	0
0	2	3	5	5	42	1	1,00	1
0	2	3	8	8	50	2	1,41	0
1	2	0	5	5	90	2	1,41	1
1	2	3	4	5	80	1	1,00	0
0	2	3	12	13	35	1	1,00	0
0	2	3	14	14	55	2	1,41	1
0	2	3	35	43	10	3	1,73	0
0	2	3	6	8	47	1	1,00	0
0	2	3	19	20	52	3	1,73	1
1	2	3	7	7	90	2	1,41	0
0	2	3	17	17	45	3	1,73	0
0	2	0	35	40	30	6	2,45	0
0	2	3	6	6	30	7	2,65	1
0	2	0	10	15	20	7	2,65	1
1	2	3	11	15	100	2	1,41	0
0	2	3	7	7	10	2	1,41	1
0	2	3	15	22	30	5	2,24	1
0	2	3	2	2	20	2	1,41	0
1	2	0	10	10	20	1	1,00	0
0	2	0	4	15	65	1	1,00	0
1	0	0	20	15	60	1	1,00	1
0	2	0	13	13	5	1	1,00	0
1	0	0	12	12	200	9	3,00	1
0	2	0	4	6	37	6	2,45	1
0	2	0	5	5	34	1	1,00	0
1	2	3	16	16	100	1	1,00	1
0	2	3	27	34	10	2	1,41	0
1	0	3	7	8	80	3	1,73	0
0	2	0	3	4	14	1	1,00	1
0	2	0	10	10	60	4	2,00	1
1	0	0	10	8	250	3	1,73	0
1	2	3	12	20	200	5	2,24	0
1	0	0	11	15	200	12	3,46	0
1	0	3	4	6	44	4	2,00	1
0	2	0	2	2	80	1	1,00	1
0	0	3	10	10	30	2	1,41	0
0	2	3	8	8	30	2	1,41	0
0	2	3	15	20	50	10	3,16	1
0	2	0	11	9	40	2	1,41	0
0	2	0	7	5	50	2	1,41	0
0	2	3	25	30	180	0	0,00	0
0	2	0	7	10	20	4	2,00	0
1	0	0	4	10	300	4	2,00	0
1	2	0	28	28	200	16	4,00	0
0	2	0	13	10	20	0	0,00	0
0	2	0	9	11	60	2	1,41	0
0	2	0	5	10	40	3	1,73	0
0	2	0	13	15	21	3	1,73	0
0	2	3	5	5	25	2	1,41	0

0	2	3	3	3	70	6	2,45	1
0	2	3	15	15	80	12	3,46	0
0	2	0	4	4	5	1	1,00	0
0	2	0	4	4	60	2	1,41	0
0	2	0	7	7	30	8	2,83	0
0	0	3	11	8	8	0	0,00	0
0	2	3	8	8	70	4	2,00	0
1	0	0	8	8	100	6	2,45	1
0	2	3	8	10	60	5	2,24	0
1	0	0	2	2	120	0	0,00	0
1	0	0	13	15	150	5	2,24	1
1	2	3	2	4	50	11	3,32	1
1	2	3	13	20	230	11	3,32	1
1	2	0	10	14	20	3	1,73	0
0	2	0	2	5	50	7	2,65	1
1	2	3	10	18	200	10	3,16	1
1	0	0	2	10	100	12	3,46	1
1	2	0	12	12	130	4	2,00	1
0	2	3	33	33	20	1	1,00	1
1	0	0	30	30	100	2	1,41	0
0	0	3	31	31	20	2	1,41	1
0	2	0	16	4	15	2	1,41	0
0	2	3	2	2	10	6	2,45	1
1	2	3	25	25	70	6	2,45	0
1	0	0	18	22	300	2	1,41	0
1	0	0	15	22	250	2	1,41	0
1	2	3	10	10	110	4	2,00	0
0	2	3	2	5	38	1	1,00	0
1	0	3	23	25	180	16	4,00	0
0	2	0	10	10	15	3	1,73	0
1	2	3	8	8	60	9	3,00	1
1	0	0	18	18	200	3	1,73	0
0	2	3	12	12	4	1	1,00	0
1	0	0	10	9	250	3	1,73	0
1	0	0	1	20	100	7	2,65	0
1	0	0	8	16	150	10	3,16	1
0	2	0	1	3	20	4	2,00	0
1	2	3	15	19	300	12	3,46	0
1	0	0	10	12	300	4	2,00	0
1	2	3	8	10	60	4	2,00	1
1	0	0	20	20	130	0	0,00	0
1	2	0	12	12	90	10	3,16	1
1	0	3	7	12	50	9	3,00	0
0	2	3	1	8	30	4	2,00	0
0	2	3	8	8	60	4	2,00	1
0	2	3	22	24	80	2	1,41	0
0	2	3	6	7	70	12	3,46	0
0	2	3	8	8	60	8	2,83	1
0	2	0	25	26	90	3	1,73	0
0	2	3	6	7	70	12	3,46	0

0	2	0	1	1	50	1	1,00	1
0	2	0	1	2	30	6	2,45	1
0	2	3	7	11	60	5	2,24	0
0	2	0	4	10	25	2	1,41	0

Imprudênc	Manutençã	Desrespeit	Condições_	Uso_de_álc	Imprudênc	Alta_Veloci	Pinos_Placa	Intervençã
2	3	4	5	0	0	0	0	0
2	0	0	0	0	0	0	0	0
0	0	0	0	0	0	0	0	0
2	0	4	0	0	7	0	0	2
0	0	0	0	0	7	8	0	0
0	0	0	5	0	0	0	0	0
0	0	0	0	0	0	0	0	0
2	0	0	0	0	0	0	0	0
0	0	4	5	6	7	8	0	0
2	0	0	0	0	0	0	0	0
0	0	0	5	0	0	0	0	0
0	0	0	0	0	0	8	0	0
2	0	0	0	0	0	8	0	0
0	0	4	5	0	7	8	1	2
2	0	4	0	0	0	8	1	2
0	0	4	5	0	7	8	1	2
2	0	0	5	0	0	8	0	0
0	0	4	5	0	7	8	0	0
0	0	4	5	0	7	8	0	0
2	0	0	5	0	0	0	0	0
0	0	4	5	0	7	0	0	0
2	0	4	5	0	7	0	0	0
2	0	4	0	6	7	8	1	2
2	0	0	0	0	0	0	0	0
2	0	0	0	0	0	0	0	0
2	0	0	5	0	0	0	0	0
2	0	0	0	0	0	0	0	0
2	0	4	0	0	0	0	0	0
2	0	4	0	0	0	0	0	0
2	0	0	0	6	0	0	0	0
2	0	0	0	0	0	0	0	0
2	0	0	0	6	7	8	1	2
2	0	4	5	0	7	8	0	0
2	0	4	0	0	7	0	1	2
2	0	4	0	0	7	0	0	0
2	0	4	0	6	7	8	1	2
2	0	4	0	0	7	0	1	2
2	0	4	0	0	7	8	0	0
2	0	4	5	0	7	8	0	0
2	0	0	5	6	7	0	1	2
2	3	4	5	0	7	8	1	2
2	0	4	5	0	7	8	0	0
2	0	4	0	6	7	8	1	2
2	0	4	0	0	7	8	0	2
2	0	4	0	6	7	8	1	2
2	0	4	5	0	7	8	0	0
2	0	0	5	0	7	8	0	0
0	0	0	0	0	0	0	0	0
0	0	0	0	0	0	0	0	0

2	0	0	0	0	0	0	0	0
2	0	0	0	0	0	0	0	0
2	0	0	5	0	0	0	0	0
2	0	0	5	0	0	0	0	0
2	0	0	0	0	0	0	0	0
0	0	0	5	0	0	0	0	0
2	0	0	5	0	0	0	1	2
2	0	0	5	0	0	8	1	2
2	0	0	0	0	0	0	0	0
2	0	0	5	0	0	0	1	2
2	0	0	5	0	0	0	0	0
2	0	0	5	0	7	0	0	0
2	0	0	5	0	0	0	0	0
2	0	0	5	0	7	0	0	0
2	0	4	0	0	0	0	0	0
2	0	0	0	0	0	0	0	0
2	0	0	5	0	0	0	1	2
2	0	0	5	0	0	0	0	0
0	0	0	0	6	7	0	0	0
2	0	0	0	0	0	0	0	0
0	0	0	0	0	0	0	0	0
0	0	0	0	0	0	0	0	0
2	0	0	0	0	0	0	0	0
2	0	0	0	0	0	0	0	0
2	0	0	5	0	0	0	0	0
2	0	0	0	0	0	0	0	0
0	0	0	0	0	0	0	0	0
2	0	0	0	0	0	0	0	0
2	0	0	0	0	0	0	0	0
0	0	0	0	0	0	0	0	0
0	0	0	0	0	0	0	1	2
2	0	0	0	0	0	0	0	0
2	0	0	0	0	0	0	1	2
2	0	0	0	0	0	0	1	2
2	0	0	0	0	0	8	1	0
0	0	0	0	0	0	0	0	0
2	0	0	5	0	0	0	0	0
2	0	0	0	0	0	0	0	0
2	0	0	5	0	0	0	1	2
2	0	0	0	0	0	0	0	0
2	0	0	5	0	0	0	0	0
0	0	0	0	0	0	0	0	0
2	0	4	5	0	0	0	0	0
2	0	4	5	0	0	0	0	0
2	3	0	0	0	0	8	0	0
0	0	0	0	0	0	0	0	0
0	0	0	5	0	7	0	0	0
2	0	0	5	0	7	0	0	0
2	0	0	5	0	0	0	0	0
2	0	0	0	0	0	0	0	0

0	0	0	0	0	7	0	0	0
2	0	0	0	0	0	0	0	0
2	0	0	0	0	0	0	0	0
2	0	0	5	0	0	0	0	0
2	0	4	0	6	7	8	0	0
0	0	0	0	0	0	0	0	0
2	0	4	5	0	7	0	0	0
0	0	0	5	0	7	8	0	0
0	0	4	0	6	7	8	0	0
0	0	0	0	0	0	0	0	0
2	0	0	5	6	7	8	0	0
2	0	0	0	0	7	0	0	0
0	0	4	5	0	7	0	0	0
0	0	0	5	0	0	0	0	0
0	0	4	5	0	7	0	0	0
2	0	4	5	0	0	0	0	0
0	0	4	5	0	7	0	0	0
2	0	0	0	0	0	0	0	0
0	3	0	5	0	0	0	0	0
2	3	0	5	0	0	0	1	2
0	0	0	5	0	0	0	0	0
2	0	0	0	0	7	0	0	0
0	0	4	0	6	7	0	0	0
0	0	0	0	0	7	0	1	2
2	0	0	0	0	0	0	0	0
2	0	0	5	0	0	0	0	0
0	0	0	5	0	0	0	0	2
2	0	0	0	0	0	0	0	0
2	0	0	0	6	0	0	1	2
2	0	4	5	0	0	0	0	0
0	0	0	0	0	7	0	0	0
2	0	0	0	0	0	0	1	2
0	0	0	5	0	0	0	0	0
2	0	0	0	0	0	0	0	0
2	0	0	0	0	0	0	1	0
0	0	4	0	0	7	8	1	2
2	0	0	0	6	7	8	0	0
2	0	0	0	0	7	0	0	0
2	0	0	5	0	0	0	1	0
2	0	0	0	6	7	0	0	0
0	0	0	0	0	0	0	0	0
0	0	0	5	0	0	8	0	0
2	0	0	5	0	7	8	1	2
2	0	0	5	0	0	0	0	0
2	0	0	5	0	7	8	0	0
2	0	0	0	0	0	0	0	2
2	0	4	5	0	7	8	0	0
2	0	0	5	0	7	8	0	0
2	0	0	0	0	7	0	0	2
2	0	4	5	0	7	8	0	0

2	0	0	0	0	0	0	0	0
2	0	0	5	0	0	0	0	0
2	0	4	5	0	0	8	0	0
2	0	0	0	0	0	8	0	0

Escoriaçõe:	Afastamen	Estado_de_	Internação	Fratura	Lesões_na_	BN.1_Excit	BN.2_Extra	BN.3_Impu
3	4	0	6	7	0	5,00	4,00	6,00
0	4	0	0	0	0	4,00	4,00	9,00
0	0	0	0	0	0	8,00	4,00	4,00
0	4	0	0	7	0	7,00	4,00	4,00
3	0	0	0	0	0	8,00	8,00	8,00
3	0	0	0	0	0	6,00	5,00	4,00
3	0	0	0	0	0	5,00	4,00	6,00
3	0	0	0	0	0	6,00	5,00	5,00
3	0	0	0	7	0	8,00	5,00	7,00
3	4	0	0	7	0	5,00	6,00	6,00
3	0	0	0	0	0	6,00	5,00	4,00
3	4	0	0	7	8	5,00	4,00	4,00
3	0	0	0	0	0	4,00	2,00	4,00
3	4	0	6	7	0	9,00	7,00	6,00
3	0	0	6	7	0	9,00	8,00	2,00
3	4	0	6	7	0	8,00	7,00	4,00
0	4	0	0	7	0	7,00	8,00	6,00
3	4	0	6	7	0	10,00	8,00	7,00
3	4	0	0	7	0	5,00	6,00	7,00
3	0	0	0	0	0	0,00	3,00	4,00
0	0	0	0	7	0	9,00	4,00	8,00
3	0	0	0	0	0	9,00	9,00	8,00
3	0	0	6	7	0	8,00	7,00	1,00
0	4	0	0	7	0	6,00	3,00	2,00
3	0	0	0	0	0	9,00	2,00	3,00
0	4	0	0	7	0	9,00	6,00	6,00
0	0	0	0	0	8	3,00	3,00	9,00
3	0	0	0	0	0	8,00	4,00	9,00
3	0	0	0	0	0	4,00	2,00	0,00
3	0	0	0	0	0	7,00	2,00	6,00
3	0	0	0	0	0	6,00	3,00	7,00
3	0	0	6	0	0	9,00	10,00	6,00
3	4	0	0	7	0	7,00	3,00	5,00
3	0	0	6	7	0	5,00	5,00	5,00
3	4	0	0	0	0	3,00	3,00	4,00
3	0	0	6	7	0	8,00	6,00	6,00
3	0	0	6	7	0	7,00	4,00	6,00
3	0	0	0	7	0	11,00	9,00	4,00
3	4	0	0	7	0	6,00	8,00	7,00
3	0	0	6	7	0	8,00	6,00	3,00
3	4	0	6	7	0	10,00	10,00	5,00
3	4	0	0	7	0	8,00	6,00	6,00
3	4	0	6	7	0	9,00	8,00	3,00
3	4	5	6	7	0	9,00	8,00	6,00
3	4	0	6	7	8	9,00	8,00	4,00
3	0	0	0	0	0	7,00	6,00	5,00
3	0	0	6	7	0	9,00	9,00	5,00
3	0	0	0	0	0	8,00	8,00	6,00
0	0	0	0	0	0	1,00	3,00	3,00

3	0	0	0	0	0	5,00	2,00	4,00
3	0	0	0	0	0	7,00	3,00	2,00
3	4	0	0	7	0	2,00	2,00	0,00
3	4	0	6	0	0	5,00	3,00	2,00
3	0	0	0	0	0	5,00	4,00	3,00
3	0	0	0	0	0	3,00	5,00	4,00
3	0	0	6	7	0	8,00	1,00	3,00
0	4	0	0	7	0	9,00	3,00	6,00
3	0	0	0	0	0	0,00	3,00	3,00
3	4	0	6	7	0	5,00	2,00	4,00
3	4	0	0	0	0	6,00	4,00	1,00
3	4	0	0	7	0	5,00	3,00	4,00
3	0	0	0	7	0	5,00	6,00	6,00
3	4	0	6	0	0	5,00	5,00	3,00
0	4	0	0	7	0	6,00	2,00	2,00
3	0	0	0	0	0	2,00	2,00	0,00
3	0	0	6	7	0	8,00	6,00	5,00
3	4	0	0	0	0	7,00	2,00	2,00
3	0	0	0	0	0	7,00	3,00	2,00
3	0	0	0	0	0	6,00	3,00	7,00
3	0	0	0	0	0	4,00	1,00	6,00
3	0	0	0	0	0	8,00	3,00	2,00
3	0	0	0	0	0	5,00	2,00	4,00
0	4	0	0	0	0	4,00	3,00	3,00
3	4	0	0	7	0	6,00	4,00	9,00
3	0	0	0	0	0	5,00	6,00	5,00
3	4	0	0	7	0	9,00	8,00	7,00
3	0	0	0	0	0	9,00	3,00	4,00
3	0	0	0	7	0	9,00	2,00	2,00
0	0	0	0	0	0	4,00	8,00	1,00
0	0	0	6	7	0	7,00	4,00	6,00
3	0	0	0	0	0	6,00	4,00	1,00
3	0	0	6	7	0	4,00	4,00	5,00
3	0	0	6	0	0	8,00	6,00	6,00
3	0	0	6	7	0	6,00	1,00	4,00
3	0	0	0	0	0	3,00	5,00	1,00
3	0	0	0	0	0	6,00	3,00	5,00
3	0	0	0	0	0	5,00	4,00	2,00
3	0	0	6	7	0	5,00	8,00	3,00
3	0	0	0	0	0	6,00	6,00	4,00
3	0	0	0	0	0	7,00	4,00	1,00
0	0	0	0	0	0	3,00	5,00	4,00
3	0	0	0	0	0	7,00	2,00	1,00
3	0	0	6	7	0	3,00	4,00	6,00
3	4	0	6	7	0	6,00	4,00	3,00
0	0	0	0	0	0	8,00	6,00	5,00
3	0	0	0	0	0	5,00	2,00	4,00
3	0	0	0	0	0	7,00	5,00	5,00
3	0	0	0	0	0	8,00	3,00	6,00
3	0	0	0	0	0	6,00	4,00	5,00

3	0	0	0	0	0	8,00	7,00	9,00
3	0	0	0	0	0	9,00	10,00	5,00
0	4	5	6	7	0	7,00	2,00	6,00
3	0	0	0	0	0	7,00	2,00	5,00
0	4	0	6	7	0	8,00	7,00	6,00
0	0	0	0	0	0	5,00	5,00	6,00
3	0	0	0	7	0	10,00	10,00	3,00
3	4	0	6	0	0	9,00	7,00	6,00
0	4	0	0	7	0	11,00	9,00	3,00
0	0	0	0	0	0	3,00	4,00	4,00
3	4	0	6	7	0	9,00	8,00	8,00
3	0	0	0	0	0	6,00	8,00	8,00
3	0	0	0	0	0	3,00	2,00	7,00
3	0	0	0	0	0	7,00	4,00	5,00
0	4	0	0	7	0	6,00	5,00	4,00
3	0	0	0	0	0	7,00	1,00	9,00
3	0	0	0	0	0	4,00	7,00	6,00
0	4	0	0	7	0	3,00	5,00	6,00
3	0	0	0	0	0	8,00	3,00	1,00
3	4	0	6	7	0	3,00	3,00	4,00
3	0	0	0	0	0	9,00	3,00	4,00
3	0	0	0	0	0	6,00	2,00	7,00
3	4	0	0	0	0	9,00	9,00	9,00
0	0	0	6	7	0	7,00	6,00	3,00
0	0	0	0	7	0	4,00	3,00	8,00
3	0	0	0	0	0	4,00	4,00	6,00
3	4	0	0	7	0	6,00	3,00	7,00
3	0	0	0	0	0	8,00	3,00	3,00
0	4	0	6	7	0	6,00	4,00	5,00
3	0	0	0	0	0	8,00	5,00	5,00
3	0	0	0	7	0	8,00	4,00	5,00
3	0	0	6	7	0	5,00	2,00	4,00
3	0	0	0	0	0	5,00	3,00	3,00
3	0	0	0	0	0	3,00	3,00	5,00
3	0	0	0	0	0	7,00	4,00	5,00
0	0	0	0	7	0	8,00	3,00	3,00
0	4	0	0	0	0	9,00	0,00	6,00
0	4	0	0	0	0	7,00	5,00	4,00
0	4	0	0	7	0	7,00	3,00	2,00
3	0	0	0	0	0	6,00	6,00	7,00
0	0	0	0	0	0	8,00	0,00	4,00
3	4	0	0	7	0	7,00	4,00	5,00
3	4	0	6	7	0	11,00	9,00	2,00
3	0	0	0	0	0	5,00	8,00	8,00
3	0	0	0	0	0	3,00	6,00	5,00
0	4	0	6	0	0	3,00	6,00	5,00
3	4	0	0	7	0	10,00	9,00	2,00
3	0	0	0	0	0	7,00	5,00	2,00
0	0	0	6	0	0	7,00	0,00	2,00
3	4	0	0	7	0	10,00	10,00	3,00

3	4	0	0	0	0	7,00	5,00	2,00
3	4	0	0	7	0	7,00	0,00	2,00
3	4	0	0	0	0	9,00	10,00	3,00
3	0	0	0	0	0	5,00	4,00	4,00

BN.4_Deso	BN.Total	ED_1	ED_2	ED_3	ED_4	ED.Total	DG_1	DG_3
5,00	20	5,00	4,00	2,00	1,00	12	8,00	3,00
4,00	21	6,00	4,00	5,00	5,00	20	7,00	5,00
6,00	22	7,00	5,00	4,00	1,00	17	7,00	2,00
7,00	22	4,00	4,00	3,00	1,00	12	4,00	5,00
7,00	31	6,00	3,00	7,00	4,00	20	5,00	6,00
4,00	19	4,00	2,00	4,00	2,00	12	4,00	8,00
6,00	21	4,00	3,00	3,00	5,00	15	5,00	4,00
4,00	20	3,00	3,00	4,00	1,00	11	6,00	2,00
3,00	23	4,00	4,00	4,00	1,00	13	6,00	4,00
6,00	23	5,00	3,00	7,00	3,00	18	9,00	6,00
3,00	18	4,00	2,00	7,00	3,00	16	8,00	1,00
4,00	17	10,00	5,00	3,00	7,00	25	5,00	6,00
3,00	13	3,00	4,00	3,00	0,00	10	3,00	3,00
6,00	28	1,00	1,00	1,00	3,00	6	3,00	6,00
7,00	26	1,00	1,00	0,00	0,00	2	4,00	7,00
6,00	25	1,00	2,00	0,00	4,00	7	2,00	5,00
9,00	30	3,00	3,00	2,00	4,00	12	2,00	5,00
7,00	32	1,00	0,00	0,00	0,00	1	4,00	4,00
7,00	25	4,00	1,00	2,00	3,00	10	6,00	6,00
4,00	11	5,00	3,00	1,00	5,00	14	8,00	6,00
4,00	25	4,00	5,00	5,00	1,00	15	5,00	6,00
6,00	32	0,00	2,00	1,00	1,00	4	7,00	5,00
8,00	24	2,00	2,00	2,00	1,00	7	1,00	3,00
5,00	16	5,00	6,00	2,00	4,00	17	2,00	7,00
2,00	16	4,00	3,00	0,00	0,00	7	7,00	7,00
3,00	24	1,00	1,00	2,00	1,00	5	7,00	5,00
2,00	17	9,00	7,00	6,00	6,00	28	9,00	4,00
6,00	27	4,00	6,00	3,00	2,00	15	5,00	5,00
4,00	10	6,00	4,00	5,00	2,00	17	5,00	4,00
4,00	19	3,00	5,00	7,00	0,00	15	5,00	2,00
6,00	22	6,00	4,00	5,00	4,00	19	8,00	7,00
5,00	30	1,00	5,00	1,00	5,00	12	4,00	6,00
4,00	19	5,00	4,00	3,00	2,00	14	8,00	5,00
4,00	19	5,00	3,00	1,00	3,00	12	6,00	2,00
5,00	15	5,00	6,00	5,00	1,00	17	2,00	1,00
6,00	26	2,00	4,00	1,00	2,00	9	3,00	5,00
5,00	22	4,00	2,00	4,00	4,00	14	4,00	4,00
7,00	31	3,00	3,00	3,00	0,00	9	4,00	4,00
6,00	27	4,00	3,00	4,00	3,00	14	7,00	8,00
5,00	22	3,00	5,00	1,00	2,00	11	9,00	3,00
7,00	32	4,00	2,00	2,00	0,00	8	6,00	5,00
5,00	25	2,00	2,00	1,00	5,00	10	5,00	6,00
6,00	26	2,00	2,00	0,00	1,00	5	4,00	6,00
5,00	28	1,00	3,00	1,00	4,00	9	4,00	5,00
8,00	29	4,00	3,00	1,00	4,00	12	4,00	6,00
3,00	21	6,00	3,00	3,00	8,00	20	3,00	1,00
7,00	30	2,00	1,00	1,00	1,00	5	5,00	4,00
4,00	26	5,00	4,00	3,00	1,00	13	3,00	4,00
4,00	11	6,00	7,00	5,00	6,00	24	6,00	4,00

5,00	16	5,00	6,00	8,00	4,00	23	4,00	3,00
4,00	16	8,00	4,00	6,00	4,00	22	6,00	2,00
2,00	6	4,00	7,00	6,00	0,00	17	4,00	4,00
1,00	11	6,00	6,00	6,00	3,00	21	5,00	3,00
3,00	15	5,00	5,00	6,00	4,00	20	4,00	4,00
3,00	15	4,00	5,00	5,00	4,00	18	2,00	3,00
2,00	14	4,00	6,00	7,00	5,00	22	6,00	5,00
3,00	21	6,00	4,00	8,00	6,00	24	7,00	5,00
4,00	10	6,00	6,00	5,00	6,00	23	6,00	4,00
3,00	14	5,00	5,00	5,00	4,00	19	6,00	4,00
3,00	14	3,00	5,00	6,00	6,00	20	6,00	3,00
4,00	16	7,00	5,00	4,00	3,00	19	6,00	4,00
5,00	22	4,00	2,00	2,00	0,00	8	1,00	5,00
5,00	18	4,00	2,00	3,00	3,00	12	8,00	5,00
2,00	12	6,00	6,00	7,00	2,00	21	5,00	4,00
2,00	6	5,00	7,00	6,00	0,00	18	4,00	4,00
6,00	25	7,00	3,00	4,00	4,00	18	9,00	8,00
3,00	14	1,00	7,00	4,00	4,00	16	5,00	2,00
4,00	16	8,00	4,00	6,00	4,00	22	6,00	2,00
4,00	20	7,00	5,00	2,00	5,00	19	10,00	6,00
3,00	14	6,00	6,00	6,00	7,00	25	7,00	4,00
3,00	16	4,00	6,00	3,00	3,00	16	6,00	5,00
5,00	16	5,00	6,00	8,00	4,00	23	4,00	3,00
4,00	14	4,00	6,00	3,00	4,00	17	3,00	2,00
6,00	25	8,00	7,00	6,00	2,00	23	6,00	5,00
2,00	18	6,00	6,00	5,00	5,00	22	6,00	3,00
3,00	27	5,00	6,00	5,00	2,00	18	8,00	7,00
3,00	19	2,00	0,00	2,00	0,00	4	3,00	8,00
7,00	20	4,00	5,00	5,00	1,00	15	7,00	5,00
8,00	21	1,00	2,00	4,00	0,00	7	2,00	3,00
5,00	22	6,00	6,00	3,00	6,00	21	5,00	7,00
3,00	14	3,00	5,00	6,00	6,00	20	6,00	3,00
6,00	19	2,00	4,00	4,00	6,00	16	4,00	6,00
6,00	26	2,00	3,00	1,00	2,00	8	3,00	5,00
2,00	13	6,00	4,00	4,00	3,00	17	4,00	4,00
2,00	11	3,00	4,00	4,00	1,00	12	7,00	5,00
5,00	19	4,00	2,00	4,00	4,00	14	4,00	4,00
3,00	14	7,00	6,00	5,00	1,00	19	9,00	5,00
3,00	19	5,00	6,00	2,00	3,00	16	10,00	3,00
3,00	19	3,00	1,00	1,00	1,00	6	1,00	2,00
1,00	13	3,00	5,00	5,00	2,00	15	7,00	4,00
2,00	14	5,00	6,00	5,00	4,00	20	3,00	3,00
4,00	14	4,00	6,00	3,00	2,00	15	9,00	6,00
6,00	19	4,00	5,00	3,00	4,00	16	7,00	5,00
6,00	19	3,00	5,00	4,00	1,00	13	7,00	5,00
3,00	22	3,00	6,00	4,00	4,00	17	4,00	7,00
2,00	13	5,00	5,00	6,00	3,00	19	4,00	3,00
4,00	21	3,00	4,00	4,00	0,00	11	2,00	4,00
2,00	19	2,00	5,00	1,00	1,00	9	2,00	6,00
5,00	20	4,00	3,00	1,00	4,00	12	6,00	3,00

4,00	28	4,00	5,00	5,00	1,00	15	6,00	7,00
8,00	32	3,00	3,00	4,00	4,00	14	7,00	6,00
4,00	19	0,00	4,00	3,00	0,00	7	4,00	5,00
5,00	19	8,00	5,00	2,00	2,00	17	7,00	6,00
6,00	27	1,00	1,00	0,00	3,00	5	3,00	6,00
2,00	18	4,00	6,00	8,00	3,00	21	3,00	3,00
7,00	30	5,00	1,00	2,00	0,00	8	7,00	5,00
6,00	28	5,00	4,00	2,00	3,00	14	8,00	4,00
7,00	30	4,00	3,00	3,00	0,00	10	5,00	4,00
5,00	16	2,00	6,00	3,00	2,00	13	4,00	2,00
4,00	29	5,00	3,00	7,00	1,00	16	7,00	5,00
7,00	29	4,00	2,00	4,00	3,00	13	7,00	8,00
6,00	18	5,00	5,00	5,00	3,00	18	4,00	3,00
4,00	20	6,00	5,00	8,00	2,00	21	6,00	5,00
5,00	20	4,00	5,00	5,00	4,00	18	6,00	5,00
3,00	20	5,00	4,00	5,00	2,00	16	7,00	6,00
5,00	22	5,00	2,00	5,00	2,00	14	6,00	4,00
3,00	17	0,00	3,00	1,00	6,00	10	4,00	1,00
2,00	14	4,00	4,00	3,00	0,00	11	5,00	5,00
4,00	14	6,00	2,00	2,00	6,00	16	7,00	4,00
1,00	17	3,00	4,00	3,00	4,00	14	2,00	8,00
8,00	23	7,00	4,00	7,00	3,00	21	7,00	0,00
4,00	31	4,00	6,00	5,00	3,00	18	8,00	8,00
5,00	21	3,00	5,00	1,00	1,00	10	9,00	4,00
6,00	21	3,00	5,00	6,00	5,00	19	5,00	3,00
2,00	16	5,00	6,00	4,00	3,00	18	7,00	6,00
4,00	20	7,00	2,00	6,00	1,00	16	4,00	3,00
2,00	16	4,00	4,00	3,00	0,00	11	7,00	6,00
4,00	19	1,00	4,00	3,00	3,00	11	5,00	2,00
7,00	25	4,00	3,00	5,00	7,00	19	3,00	6,00
3,00	20	2,00	3,00	5,00	4,00	14	7,00	5,00
3,00	14	5,00	5,00	5,00	4,00	19	6,00	4,00
3,00	14	7,00	5,00	5,00	3,00	20	6,00	3,00
4,00	15	3,00	5,00	7,00	3,00	18	7,00	3,00
6,00	22	3,00	7,00	5,00	1,00	16	5,00	4,00
5,00	19	5,00	5,00	2,00	4,00	16	3,00	2,00
4,00	19	3,00	2,00	3,00	3,00	11	4,00	8,00
3,00	19	4,00	4,00	1,00	2,00	11	8,00	6,00
2,00	14	4,00	2,00	5,00	0,00	11	5,00	4,00
5,00	24	2,00	3,00	2,00	6,00	13	8,00	6,00
3,00	15	4,00	6,00	6,00	4,00	20	6,00	6,00
4,00	20	4,00	7,00	5,00	4,00	20	7,00	6,00
8,00	30	1,00	1,00	2,00	1,00	5	2,00	6,00
7,00	28	5,00	3,00	6,00	6,00	20	3,00	2,00
7,00	21	6,00	5,00	8,00	6,00	25	5,00	0,00
7,00	21	6,00	5,00	8,00	6,00	25	5,00	0,00
6,00	27	5,00	2,00	2,00	0,00	9	7,00	5,00
7,00	21	6,00	4,00	4,00	5,00	19	7,00	3,00
5,00	14	8,00	5,00	5,00	1,00	19	5,00	4,00
7,00	30	5,00	1,00	2,00	0,00	8	7,00	5,00

7,00	21	6,00	4,00	4,00	5,00	19	7,00	3,00
5,00	14	8,00	5,00	5,00	1,00	19	5,00	4,00
7,00	29	5,00	1,00	2,00	0,00	8	7,00	5,00
3,00	16	6,00	5,00	6,00	5,00	22	4,00	4,00

DG_4	DG.Total	DG2.PERSIS	AD_1	AD_2	AD_3	AD_4	AD_5	AD.Total
1,00	12	5	3,00	5,00	2,00	5,00	8,00	23
2,00	14	5	2,00	5,00	0,00	4,00	8,00	19
3,00	12	6	5,00	6,00	2,00	4,00	5,00	22
5,00	14	7	6,00	7,00	5,00	6,00	6,00	30
2,00	13	4	4,00	2,00	3,00	1,00	8,00	18
3,00	15	5	5,00	5,00	5,00	4,00	11,00	30
4,00	13	4	4,00	6,00	1,00	5,00	7,00	23
2,00	10	6	7,00	7,00	5,00	7,00	10,00	36
3,00	13	7	8,00	8,00	4,00	5,00	11,00	36
2,00	17	5	1,00	4,00	2,00	3,00	6,00	16
2,00	11	7	8,00	5,00	4,00	3,00	12,00	32
2,00	13	7	5,00	4,00	4,00	7,00	11,00	31
4,00	10	5	8,00	7,00	5,00	8,00	12,00	40
2,00	11	7	6,00	7,00	5,00	11,00	11,00	40
3,00	14	8	2,00	5,00	4,00	5,00	5,00	21
1,00	8	6	6,00	7,00	5,00	8,00	11,00	37
2,00	9	4	6,00	4,00	4,00	2,00	7,00	23
3,00	11	7	4,00	5,00	5,00	7,00	7,00	28
4,00	16	6	3,00	7,00	2,00	7,00	7,00	26
3,00	17	4	4,00	5,00	3,00	4,00	5,00	21
4,00	15	7	2,00	5,00	3,00	7,00	8,00	25
2,00	14	2	6,00	5,00	5,00	4,00	8,00	28
3,00	7	8	3,00	4,00	4,00	4,00	3,00	18
2,00	11	3	5,00	7,00	4,00	9,00	9,00	34
5,00	19	8	8,00	8,00	5,00	4,00	12,00	37
3,00	15	5	8,00	6,00	5,00	5,00	12,00	36
4,00	17	4	5,00	3,00	2,00	9,00	6,00	25
4,00	14	4	7,00	5,00	4,00	6,00	10,00	32
3,00	12	7	4,00	6,00	2,00	1,00	9,00	22
2,00	9	7	6,00	5,00	4,00	8,00	11,00	34
4,00	19	7	6,00	7,00	4,00	4,00	10,00	31
2,00	12	6	7,00	5,00	4,00	8,00	9,00	33
1,00	14	5	3,00	5,00	2,00	6,00	5,00	21
1,00	9	6	5,00	4,00	1,00	5,00	6,00	21
2,00	5	7	8,00	7,00	4,00	10,00	9,00	38
1,00	9	6	6,00	6,00	5,00	8,00	10,00	35
1,00	9	6	4,00	5,00	3,00	2,00	10,00	24
3,00	11	7	2,00	6,00	4,00	7,00	4,00	23
2,00	17	5	7,00	8,00	4,00	5,00	11,00	35
3,00	15	5	5,00	5,00	3,00	5,00	7,00	25
3,00	14	7	4,00	3,00	5,00	4,00	5,00	21
3,00	14	6	6,00	5,00	5,00	8,00	10,00	34
3,00	13	8	4,00	5,00	5,00	4,00	7,00	25
1,00	10	7	7,00	6,00	4,00	8,00	9,00	34
4,00	14	5	6,00	4,00	3,00	6,00	8,00	27
4,00	8	6	4,00	6,00	3,00	5,00	8,00	26
4,00	13	6	4,00	4,00	5,00	5,00	8,00	26
5,00	12	6	8,00	6,00	3,00	4,00	9,00	30
4,00	14	8	3,00	5,00	0,00	5,00	10,00	23

2,00	9	8	3,00	4,00	2,00	9,00	9,00	27
2,00	10	6	1,00	3,00	2,00	6,00	9,00	21
2,00	10	8	7,00	7,00	4,00	10,00	9,00	37
6,00	14	5	3,00	7,00	2,00	4,00	11,00	27
3,00	11	6	4,00	6,00	1,00	4,00	6,00	21
5,00	10	7	6,00	6,00	4,00	6,00	10,00	32
4,00	15	3	6,00	8,00	3,00	9,00	10,00	36
3,00	15	6	6,00	8,00	5,00	8,00	9,00	36
3,00	13	8	3,00	4,00	0,00	5,00	10,00	22
2,00	12	6	6,00	7,00	3,00	9,00	10,00	35
2,00	11	6	5,00	5,00	5,00	8,00	11,00	34
2,00	12	7	6,00	7,00	2,00	5,00	8,00	28
3,00	9	5	7,00	6,00	5,00	9,00	10,00	37
2,00	15	7	3,00	8,00	2,00	3,00	7,00	23
6,00	15	6	3,00	8,00	2,00	4,00	10,00	27
2,00	10	8	6,00	8,00	4,00	10,00	9,00	37
4,00	21	2	3,00	4,00	3,00	5,00	5,00	20
3,00	10	7	6,00	7,00	3,00	2,00	11,00	29
2,00	10	6	1,00	3,00	2,00	6,00	9,00	21
3,00	19	6	2,00	1,00	3,00	3,00	7,00	16
4,00	15	5	5,00	5,00	4,00	10,00	7,00	31
4,00	15	5	7,00	7,00	5,00	7,00	7,00	33
2,00	9	8	3,00	4,00	2,00	9,00	9,00	27
2,00	7	7	5,00	5,00	5,00	8,00	9,00	32
3,00	14	2	7,00	6,00	1,00	6,00	8,00	28
2,00	11	7	5,00	3,00	2,00	8,00	8,00	26
2,00	17	7	6,00	5,00	5,00	7,00	11,00	34
3,00	14	8	7,00	8,00	4,00	8,00	11,00	38
2,00	14	8	7,00	8,00	4,00	5,00	10,00	34
0,00	5	6	8,00	6,00	4,00	6,00	8,00	32
2,00	14	5	4,00	4,00	3,00	3,00	8,00	22
2,00	11	6	5,00	5,00	5,00	8,00	11,00	34
3,00	13	2	3,00	6,00	3,00	8,00	8,00	28
2,00	10	6	6,00	6,00	5,00	9,00	11,00	37
2,00	10	5	6,00	8,00	5,00	11,00	10,00	40
2,00	14	7	6,00	5,00	4,00	6,00	11,00	32
2,00	10	6	5,00	4,00	3,00	2,00	10,00	24
4,00	18	6	5,00	5,00	5,00	4,00	10,00	29
2,00	15	4	3,00	6,00	4,00	5,00	7,00	25
1,00	4	8	7,00	7,00	5,00	5,00	12,00	36
4,00	15	7	8,00	8,00	5,00	10,00	12,00	43
5,00	11	6	7,00	6,00	4,00	7,00	10,00	34
3,00	18	8	6,00	8,00	5,00	6,00	12,00	37
2,00	14	4	3,00	7,00	2,00	3,00	9,00	24
5,00	17	6	8,00	8,00	5,00	11,00	12,00	44
4,00	15	7	8,00	6,00	5,00	7,00	11,00	37
5,00	12	6	5,00	6,00	4,00	7,00	11,00	33
2,00	8	4	8,00	7,00	2,00	4,00	9,00	30
4,00	12	8	7,00	7,00	5,00	8,00	12,00	39
1,00	10	5	4,00	5,00	2,00	5,00	9,00	25

6,00	19	4	6,00	8,00	4,00	6,00	9,00	33
3,00	16	1	5,00	5,00	4,00	2,00	7,00	23
5,00	14	8	8,00	8,00	5,00	1,00	10,00	32
5,00	18	7	5,00	6,00	4,00	3,00	10,00	28
2,00	11	7	7,00	7,00	5,00	11,00	11,00	41
2,00	8	8	5,00	6,00	5,00	8,00	10,00	34
4,00	16	7	3,00	4,00	5,00	4,00	5,00	21
2,00	14	8	2,00	4,00	1,00	5,00	6,00	18
3,00	12	7	3,00	6,00	4,00	7,00	4,00	24
1,00	7	4	4,00	4,00	3,00	5,00	7,00	23
3,00	15	6	5,00	6,00	3,00	8,00	6,00	28
2,00	17	5	7,00	8,00	4,00	4,00	10,00	33
3,00	10	6	7,00	6,00	2,00	7,00	5,00	27
2,00	13	8	5,00	7,00	5,00	5,00	11,00	33
2,00	13	6	6,00	6,00	4,00	8,00	11,00	35
5,00	18	6	7,00	7,00	4,00	5,00	9,00	32
3,00	13	4	4,00	5,00	1,00	5,00	5,00	20
3,00	8	6	6,00	1,00	2,00	7,00	10,00	26
4,00	14	4	8,00	6,00	4,00	9,00	11,00	38
2,00	13	5	4,00	4,00	2,00	5,00	6,00	21
3,00	13	3	8,00	7,00	5,00	10,00	12,00	42
5,00	12	4	3,00	5,00	2,00	2,00	9,00	21
2,00	18	7	5,00	6,00	5,00	2,00	9,00	27
3,00	16	4	4,00	5,00	3,00	6,00	7,00	25
3,00	11	5	2,00	3,00	3,00	4,00	8,00	20
4,00	17	6	6,00	7,00	4,00	7,00	10,00	34
2,00	9	8	5,00	8,00	4,00	5,00	9,00	31
2,00	15	6	7,00	8,00	4,00	2,00	9,00	30
5,00	12	6	7,00	5,00	4,00	7,00	8,00	31
2,00	11	5	3,00	4,00	3,00	6,00	6,00	22
3,00	15	4	4,00	8,00	5,00	7,00	11,00	35
2,00	12	6	6,00	7,00	3,00	9,00	10,00	35
3,00	12	6	6,00	8,00	1,00	4,00	8,00	27
0,00	10	5	3,00	5,00	4,00	8,00	7,00	27
3,00	12	6	5,00	6,00	1,00	10,00	8,00	30
1,00	6	6	6,00	4,00	4,00	3,00	9,00	26
4,00	16	7	7,00	7,00	5,00	7,00	9,00	35
4,00	18	7	5,00	5,00	5,00	9,00	9,00	33
4,00	13	5	7,00	8,00	5,00	9,00	12,00	41
1,00	15	6	4,00	7,00	4,00	3,00	8,00	26
5,00	17	4	6,00	8,00	4,00	9,00	9,00	36
5,00	18	6	7,00	8,00	5,00	8,00	11,00	39
4,00	12	8	3,00	6,00	4,00	5,00	6,00	24
1,00	6	3	4,00	2,00	2,00	3,00	6,00	17
3,00	8	2	3,00	2,00	0,00	0,00	4,00	9
3,00	8	2	3,00	2,00	0,00	0,00	4,00	9
4,00	16	7	3,00	4,00	5,00	5,00	5,00	22
3,00	13	3	6,00	5,00	1,00	5,00	8,00	25
4,00	13	8	8,00	7,00	5,00	7,00	10,00	37
4,00	16	7	3,00	4,00	5,00	4,00	5,00	21

3,00	13	3	6,00	5,00	1,00	5,00	8,00	25
4,00	13	8	8,00	7,00	5,00	7,00	10,00	37
4,00	16	7	3,00	4,00	5,00	4,00	5,00	21
2,00	10	5	4,00	7,00	1,00	5,00	6,00	23

CO_1	CO_2	CO_3	CO_4	CO_5	CO.Total	AT_1	AT_2	AT_3
6,00	3,00	4,00	8,00	5,00	26	10,00	8,00	8,00
4,00	3,00	5,00	4,00	6,00	22	4,00	4,00	11,00
6,00	2,00	6,00	6,00	5,00	25	8,00	4,00	9,00
6,00	6,00	6,00	4,00	6,00	28	3,00	4,00	8,00
3,00	3,00	8,00	9,00	6,00	29	3,00	2,00	6,00
1,00	3,00	8,00	2,00	6,00	20	6,00	2,00	4,00
4,00	2,00	2,00	6,00	5,00	19	7,00	7,00	5,00
8,00	5,00	5,00	9,00	7,00	34	8,00	5,00	8,00
8,00	2,00	7,00	8,00	9,00	34	2,00	1,00	4,00
6,00	4,00	5,00	9,00	6,00	30	10,00	6,00	10,00
6,00	5,00	5,00	6,00	8,00	30	11,00	5,00	12,00
8,00	7,00	7,00	9,00	9,00	40	7,00	2,00	11,00
6,00	5,00	6,00	7,00	8,00	32	5,00	6,00	10,00
8,00	5,00	6,00	9,00	7,00	35	8,00	2,00	9,00
5,00	5,00	6,00	1,00	6,00	23	7,00	5,00	11,00
7,00	6,00	5,00	9,00	6,00	33	9,00	4,00	8,00
2,00	1,00	4,00	6,00	4,00	17	8,00	6,00	8,00
1,00	3,00	6,00	0,00	6,00	16	10,00	5,00	12,00
3,00	4,00	4,00	6,00	3,00	20	7,00	4,00	4,00
3,00	4,00	5,00	8,00	6,00	26	7,00	7,00	4,00
6,00	5,00	5,00	3,00	6,00	25	7,00	6,00	10,00
7,00	5,00	7,00	9,00	7,00	35	10,00	8,00	11,00
2,00	5,00	5,00	0,00	6,00	18	7,00	2,00	9,00
6,00	4,00	5,00	6,00	7,00	28	3,00	2,00	0,00
7,00	7,00	7,00	10,00	8,00	39	9,00	5,00	6,00
8,00	5,00	7,00	9,00	8,00	37	7,00	7,00	9,00
8,00	6,00	6,00	10,00	8,00	38	9,00	3,00	3,00
8,00	5,00	6,00	10,00	7,00	36	9,00	8,00	12,00
6,00	5,00	5,00	9,00	5,00	30	9,00	6,00	8,00
8,00	4,00	4,00	7,00	6,00	29	9,00	4,00	8,00
6,00	6,00	5,00	10,00	6,00	33	6,00	6,00	10,00
5,00	6,00	7,00	9,00	6,00	33	3,00	2,00	4,00
4,00	3,00	4,00	7,00	5,00	23	9,00	8,00	7,00
4,00	4,00	5,00	5,00	4,00	22	8,00	8,00	8,00
5,00	3,00	5,00	5,00	7,00	25	5,00	2,00	8,00
7,00	6,00	5,00	8,00	6,00	32	5,00	2,00	5,00
5,00	3,00	5,00	6,00	6,00	25	3,00	2,00	6,00
6,00	4,00	6,00	1,00	4,00	21	6,00	2,00	9,00
8,00	6,00	4,00	9,00	7,00	34	5,00	4,00	5,00
7,00	6,00	7,00	8,00	8,00	36	8,00	8,00	12,00
6,00	5,00	6,00	1,00	4,00	22	8,00	1,00	9,00
8,00	5,00	6,00	9,00	6,00	34	7,00	4,00	9,00
4,00	4,00	6,00	3,00	7,00	24	6,00	6,00	9,00
4,00	5,00	4,00	7,00	5,00	25	6,00	4,00	8,00
5,00	2,00	5,00	7,00	5,00	24	3,00	3,00	5,00
5,00	5,00	4,00	4,00	6,00	24	6,00	5,00	6,00
3,00	3,00	5,00	1,00	6,00	18	9,00	4,00	9,00
7,00	4,00	5,00	10,00	7,00	33	4,00	2,00	2,00
8,00	4,00	6,00	10,00	8,00	36	8,00	4,00	11,00

4,00	2,00	5,00	9,00	8,00	28	4,00	3,00	5,00
5,00	3,00	5,00	7,00	7,00	27	10,00	5,00	9,00
7,00	5,00	5,00	9,00	9,00	35	8,00	7,00	11,00
7,00	5,00	6,00	9,00	5,00	32	5,00	2,00	10,00
2,00	1,00	4,00	8,00	6,00	21	3,00	3,00	3,00
7,00	6,00	8,00	7,00	7,00	35	5,00	7,00	6,00
8,00	6,00	8,00	10,00	9,00	41	4,00	7,00	11,00
7,00	3,00	6,00	6,00	7,00	29	5,00	5,00	9,00
7,00	3,00	5,00	9,00	8,00	32	8,00	5,00	9,00
7,00	3,00	6,00	8,00	5,00	29	3,00	3,00	4,00
6,00	5,00	4,00	9,00	5,00	29	7,00	5,00	5,00
6,00	4,00	5,00	7,00	7,00	29	9,00	5,00	2,00
7,00	4,00	6,00	8,00	6,00	31	0,00	0,00	6,00
5,00	5,00	4,00	8,00	7,00	29	10,00	5,00	8,00
7,00	6,00	6,00	9,00	5,00	33	5,00	2,00	9,00
8,00	5,00	6,00	9,00	9,00	37	8,00	7,00	12,00
7,00	6,00	6,00	4,00	8,00	31	9,00	5,00	10,00
6,00	2,00	7,00	8,00	6,00	29	6,00	6,00	11,00
5,00	3,00	5,00	7,00	7,00	27	10,00	5,00	9,00
6,00	4,00	4,00	10,00	4,00	28	8,00	5,00	9,00
8,00	3,00	6,00	10,00	8,00	35	1,00	2,00	10,00
6,00	3,00	6,00	8,00	6,00	29	5,00	4,00	4,00
4,00	2,00	5,00	9,00	8,00	28	4,00	3,00	5,00
8,00	2,00	5,00	7,00	8,00	30	5,00	2,00	7,00
7,00	5,00	5,00	7,00	9,00	33	7,00	4,00	7,00
6,00	4,00	6,00	8,00	8,00	32	8,00	1,00	4,00
7,00	4,00	8,00	8,00	4,00	31	9,00	5,00	11,00
7,00	6,00	7,00	8,00	7,00	35	7,00	4,00	12,00
7,00	6,00	5,00	5,00	6,00	29	3,00	6,00	11,00
7,00	2,00	5,00	5,00	4,00	23	4,00	3,00	6,00
6,00	5,00	3,00	7,00	6,00	27	9,00	3,00	5,00
6,00	5,00	4,00	9,00	5,00	29	7,00	5,00	5,00
6,00	3,00	2,00	4,00	3,00	18	6,00	5,00	7,00
7,00	6,00	5,00	8,00	6,00	32	3,00	2,00	5,00
7,00	6,00	6,00	9,00	7,00	35	6,00	2,00	3,00
7,00	4,00	5,00	10,00	6,00	32	4,00	8,00	9,00
5,00	3,00	5,00	6,00	6,00	25	4,00	3,00	7,00
8,00	6,00	7,00	8,00	6,00	35	7,00	6,00	6,00
4,00	4,00	4,00	4,00	2,00	18	6,00	4,00	9,00
8,00	5,00	6,00	7,00	7,00	33	3,00	2,00	7,00
8,00	7,00	7,00	9,00	9,00	40	9,00	3,00	10,00
7,00	6,00	8,00	7,00	7,00	35	5,00	7,00	6,00
6,00	6,00	7,00	10,00	8,00	37	7,00	6,00	9,00
5,00	4,00	5,00	7,00	5,00	26	8,00	6,00	10,00
7,00	4,00	8,00	10,00	7,00	36	3,00	4,00	5,00
8,00	5,00	6,00	8,00	8,00	35	3,00	2,00	6,00
8,00	7,00	7,00	7,00	6,00	35	3,00	1,00	7,00
7,00	3,00	8,00	6,00	5,00	29	4,00	1,00	3,00
7,00	6,00	6,00	9,00	8,00	36	10,00	7,00	11,00
5,00	4,00	4,00	5,00	3,00	21	8,00	8,00	9,00

8,00	4,00	7,00	4,00	7,00	30	1,00	2,00	4,00
6,00	5,00	5,00	3,00	7,00	26	6,00	3,00	7,00
6,00	5,00	6,00	2,00	7,00	26	9,00	0,00	7,00
7,00	6,00	6,00	9,00	8,00	36	9,00	1,00	4,00
8,00	6,00	6,00	9,00	7,00	36	8,00	2,00	9,00
5,00	5,00	5,00	8,00	7,00	30	8,00	3,00	5,00
5,00	4,00	6,00	1,00	4,00	20	8,00	1,00	11,00
4,00	3,00	6,00	10,00	7,00	30	8,00	6,00	11,00
5,00	4,00	6,00	0,00	4,00	19	7,00	2,00	10,00
4,00	5,00	3,00	6,00	5,00	23	5,00	5,00	11,00
5,00	3,00	6,00	8,00	7,00	29	9,00	6,00	11,00
8,00	6,00	4,00	9,00	7,00	34	5,00	3,00	5,00
6,00	3,00	5,00	8,00	5,00	27	6,00	3,00	6,00
6,00	5,00	8,00	10,00	8,00	37	10,00	4,00	13,00
6,00	5,00	6,00	9,00	4,00	30	8,00	5,00	5,00
8,00	7,00	6,00	10,00	8,00	39	8,00	9,00	11,00
4,00	4,00	3,00	4,00	3,00	18	6,00	6,00	7,00
7,00	1,00	4,00	5,00	4,00	21	6,00	4,00	10,00
6,00	6,00	8,00	10,00	7,00	37	3,00	5,00	6,00
5,00	2,00	5,00	6,00	6,00	24	4,00	6,00	6,00
7,00	7,00	7,00	10,00	7,00	38	4,00	1,00	4,00
4,00	5,00	6,00	3,00	8,00	26	5,00	0,00	4,00
3,00	5,00	6,00	9,00	7,00	30	10,00	6,00	11,00
8,00	7,00	7,00	8,00	8,00	38	9,00	9,00	13,00
4,00	4,00	6,00	7,00	5,00	26	8,00	5,00	9,00
5,00	4,00	7,00	9,00	5,00	30	10,00	8,00	10,00
2,00	2,00	5,00	2,00	8,00	19	8,00	3,00	12,00
8,00	6,00	6,00	10,00	8,00	38	9,00	5,00	11,00
4,00	2,00	4,00	8,00	5,00	23	4,00	7,00	8,00
3,00	4,00	2,00	7,00	5,00	21	7,00	4,00	10,00
8,00	6,00	7,00	10,00	7,00	38	10,00	5,00	8,00
7,00	3,00	6,00	8,00	5,00	29	3,00	3,00	4,00
7,00	4,00	6,00	5,00	7,00	29	9,00	4,00	2,00
6,00	3,00	6,00	9,00	6,00	30	8,00	5,00	7,00
6,00	3,00	5,00	8,00	5,00	27	6,00	2,00	6,00
7,00	2,00	5,00	5,00	8,00	27	5,00	5,00	10,00
8,00	5,00	7,00	9,00	7,00	36	7,00	3,00	10,00
8,00	7,00	5,00	7,00	7,00	34	7,00	3,00	9,00
7,00	7,00	6,00	6,00	8,00	34	3,00	1,00	7,00
6,00	4,00	7,00	8,00	6,00	31	9,00	4,00	7,00
7,00	7,00	8,00	10,00	9,00	41	4,00	8,00	11,00
6,00	6,00	7,00	8,00	9,00	36	2,00	1,00	4,00
7,00	3,00	6,00	0,00	5,00	21	6,00	1,00	11,00
6,00	3,00	4,00	5,00	5,00	23	10,00	4,00	7,00
6,00	6,00	2,00	9,00	9,00	32	6,00	2,00	7,00
6,00	6,00	2,00	9,00	9,00	32	6,00	2,00	7,00
5,00	4,00	7,00	1,00	4,00	21	8,00	2,00	11,00
4,00	5,00	2,00	6,00	3,00	20	4,00	5,00	7,00
8,00	6,00	6,00	8,00	7,00	35	4,00	4,00	7,00
5,00	4,00	6,00	1,00	4,00	20	8,00	1,00	11,00

4,00	5,00	2,00	6,00	3,00	20	4,00	5,00	7,00
8,00	6,00	6,00	8,00	7,00	35	4,00	4,00	7,00
5,00	4,00	6,00	1,00	5,00	21	8,00	0,00	10,00
3,00	2,00	6,00	7,00	4,00	22	2,00	2,00	4,00

AT.Total	Busca_de_	Esquiva_ac	Dependênc	Persistênci	Autodirecio	Cooperativ	Autotransc	Acidentes
26	2	1	1	2	1	1	2	2
19	2	2	1	2	1	1	1	2
21	2	2	1	2	1	1	2	1
15	2	1	1	2	1	1	1	2
11	2	2	1	1	1	1	1	2
12	2	1	1	2	1	1	1	2
19	2	1	1	1	1	1	1	2
21	2	1	1	2	2	2	2	2
7	2	1	1	2	2	2	1	2
26	2	2	2	2	1	1	2	2
28	1	1	1	2	1	1	2	2
20	1	2	1	2	1	2	2	2
21	1	1	1	2	2	1	2	2
19	2	1	1	2	2	2	1	2
23	2	1	1	2	1	1	2	2
21	2	1	1	2	2	1	2	2
22	2	1	1	1	1	1	2	2
27	2	1	1	2	1	1	2	2
15	2	1	2	2	1	1	1	2
18	1	1	2	1	1	1	1	2
23	2	1	1	2	1	1	2	2
29	2	1	1	1	1	2	2	2
18	2	1	1	2	1	1	1	2
5	1	2	1	1	1	1	1	2
20	1	1	2	2	2	2	2	2
23	2	1	1	2	2	2	2	2
15	1	2	2	1	1	2	1	2
29	2	1	1	1	1	2	2	2
23	1	2	1	2	1	1	2	2
21	2	1	1	2	1	1	2	2
22	2	2	2	2	1	1	2	2
9	2	1	1	2	1	1	1	2
24	2	1	1	2	1	1	2	2
24	2	1	1	2	1	1	2	2
15	1	2	1	2	2	1	1	2
12	2	1	1	2	2	1	1	2
11	2	1	1	2	1	1	1	2
17	2	1	1	2	1	1	1	2
14	2	1	2	2	2	2	1	2
28	2	1	1	2	1	2	2	2
18	2	1	1	2	1	1	1	2
20	2	1	1	2	1	2	2	2
21	2	1	1	2	1	1	2	2
18	2	1	1	2	1	1	1	2
11	2	1	1	2	1	1	1	2
17	2	2	1	2	1	1	1	2
22	2	1	1	2	1	1	2	2
8	2	1	1	2	1	1	1	2
23	1	2	1	2	1	2	2	1

12	1	2	1	2	1	1	1	2
24	1	2	1	2	1	1	2	2
26	1	2	1	2	2	2	2	2
17	1	2	1	2	1	1	1	2
9	1	2	1	2	1	1	1	2
18	1	2	1	2	1	2	1	2
22	1	2	1	1	2	2	2	2
19	2	2	1	2	2	1	1	2
22	1	2	1	2	1	1	2	2
10	1	2	1	2	2	1	1	2
17	1	2	1	2	1	1	1	2
16	1	2	1	2	1	1	1	2
6	2	1	1	2	2	1	1	2
23	1	1	1	2	1	1	2	2
16	1	2	1	2	1	1	1	2
27	1	2	1	2	2	2	2	2
24	2	2	2	1	1	1	2	2
23	1	1	1	2	1	1	2	2
24	1	2	1	2	1	1	2	2
22	2	2	2	2	1	1	2	2
13	1	2	1	2	1	2	1	2
13	1	1	1	2	1	1	1	2
12	1	2	1	2	1	1	1	2
14	1	2	1	2	1	1	1	2
18	2	2	1	1	1	1	1	2
13	1	2	1	2	1	1	1	2
25	2	2	2	2	1	1	2	2
23	2	1	1	2	2	2	2	2
20	2	1	1	2	1	1	2	2
13	2	1	1	2	1	1	1	2
17	2	2	1	2	1	1	1	2
17	1	2	1	2	1	1	1	2
18	2	1	1	1	1	1	1	2
10	2	1	1	2	2	1	1	2
11	1	2	1	2	2	2	1	2
21	1	1	1	2	1	1	2	2
14	2	1	1	2	1	1	1	2
19	1	2	2	2	1	2	1	2
19	2	1	1	1	1	1	1	2
12	2	1	1	2	2	1	1	2
22	1	1	1	2	2	2	2	2
18	1	2	1	2	1	2	1	1
22	1	1	2	2	2	2	2	2
24	2	1	1	1	1	1	2	2
12	2	1	2	2	2	2	1	2
11	2	2	1	2	2	2	1	1
11	1	2	1	2	1	2	1	2
8	2	1	1	1	1	1	1	2
28	2	1	1	2	2	2	2	2
25	2	1	1	2	1	1	2	2

7	2	1	2	1	1	1	1	2
16	2	1	2	1	1	1	1	2
16	2	1	1	2	1	1	1	2
14	2	2	2	2	1	2	1	2
19	2	1	1	2	2	2	1	2
16	1	2	1	2	1	1	1	1
20	2	1	2	2	1	1	2	2
25	2	1	1	2	1	1	2	2
19	2	1	1	2	1	1	1	2
21	1	1	1	1	1	1	2	1
26	2	1	1	2	1	1	2	2
13	2	1	2	2	1	2	1	2
15	1	2	1	2	1	1	1	2
27	2	2	1	2	1	2	2	2
18	2	2	1	2	2	1	1	2
28	2	1	2	2	1	2	2	2
19	2	1	1	1	1	1	1	2
20	1	1	1	2	1	1	2	2
14	1	1	1	1	2	2	1	2
16	1	1	1	2	1	1	1	2
9	1	1	1	1	2	2	1	2
9	2	2	1	1	1	1	1	2
27	2	2	2	2	1	1	2	2
31	2	1	2	1	1	2	2	2
22	2	2	1	2	1	1	2	2
28	1	2	2	2	1	1	2	2
23	2	1	1	2	1	1	2	2
25	1	1	1	2	1	2	2	2
19	2	1	1	2	1	1	1	2
21	2	2	1	2	1	1	2	2
23	2	1	1	1	2	2	2	2
10	1	2	1	2	2	1	1	2
15	1	2	1	2	1	1	1	2
20	1	2	1	2	1	1	2	2
14	2	1	1	2	1	1	1	2
20	2	1	1	2	1	1	2	2
20	2	1	2	2	2	2	2	2
19	2	1	2	2	1	2	1	2
11	1	1	1	2	2	2	1	2
20	2	1	1	2	1	1	2	2
23	1	2	2	1	2	2	2	1
7	2	2	2	2	2	2	1	2
18	2	1	1	2	1	1	1	2
21	2	2	1	1	1	1	2	2
15	2	2	1	1	1	1	1	2
15	2	2	1	1	1	1	1	2
21	2	1	2	2	1	1	2	2
16	2	2	1	1	1	1	1	2
15	1	2	1	2	2	2	1	2
20	2	1	2	2	1	1	2	2

16	2	2	1	1	1	1	1	2
15	1	2	1	2	2	2	1	2
18	2	1	2	2	1	1	1	2
8	1	2	1	2	1	1	1	2

RES_1	ZRE_1	SRE_1	RES_2	ZRE_2	SRE_2
5,97	1,81	1,82	1,16	1,48	1,48
-2,16	-0,66	-0,66	-0,32	-0,41	-0,42
-5,58	-1,69	-1,71	-2,26	-2,88	-2,92
8,87	2,69	2,71	1,55	1,98	2,00
-1,84	-0,56	-0,56	-0,29	-0,37	-0,37
-2,00	-0,61	-0,61	-0,31	-0,39	-0,39
-4,90	-1,49	-1,49	-1,29	-1,65	-1,66
-2,90	-0,88	-0,88	-0,56	-0,72	-0,72
4,26	1,29	1,30	0,89	1,13	1,14
-0,90	-0,27	-0,27	-0,06	-0,07	-0,07
-1,30	-0,39	-0,40	-0,40	-0,51	-0,52
-0,69	-0,21	-0,21	-0,03	-0,04	-0,04
2,38	0,72	0,73	0,80	1,02	1,03
2,23	0,68	0,68	0,55	0,70	0,71
4,16	1,26	1,27	0,88	1,12	1,13
0,13	0,04	0,04	0,16	0,20	0,20
-1,80	-0,55	-0,55	-0,28	-0,36	-0,36
0,13	0,04	0,04	0,16	0,20	0,20
1,00	0,30	0,31	0,34	0,43	0,44
-0,13	-0,04	-0,04	0,03	0,04	0,04
-0,87	-0,26	-0,26	-0,05	-0,07	-0,07
0,87	0,26	0,27	0,33	0,42	0,42
2,13	0,65	0,65	0,54	0,69	0,69
1,57	0,48	0,48	0,59	0,75	0,75
-0,46	-0,14	-0,14	0,00	-0,01	-0,01
-3,23	-0,98	-0,99	-0,60	-0,77	-0,77
-1,88	-0,57	-0,58	-0,47	-0,60	-0,61
-2,00	-0,61	-0,61	-0,31	-0,39	-0,39
-0,49	-0,15	-0,15	-0,01	-0,01	-0,01
-4,29	-1,30	-1,31	-0,92	-1,18	-1,19
9,16	2,78	2,80	1,59	2,03	2,04
4,87	1,48	1,49	1,00	1,27	1,28
0,10	0,03	0,03	0,16	0,20	0,20
0,33	0,10	0,10	0,18	0,23	0,23
0,25	0,08	0,08	0,28	0,36	0,36
3,03	0,92	0,92	0,70	0,89	0,90
0,16	0,05	0,05	0,16	0,21	0,21
-1,87	-0,57	-0,57	-0,29	-0,37	-0,37
-0,03	-0,01	-0,01	0,14	0,18	0,18
-0,87	-0,26	-0,26	-0,05	-0,07	-0,07
2,10	0,64	0,64	0,53	0,68	0,69
-0,80	-0,24	-0,25	-0,05	-0,06	-0,06
2,20	0,67	0,67	0,55	0,70	0,70
2,07	0,63	0,63	0,53	0,68	0,68
3,03	0,92	0,92	0,70	0,89	0,90
3,23	0,98	0,99	0,72	0,92	0,93
-2,74	-0,83	-0,84	-0,54	-0,70	-0,70
-4,10	-1,24	-1,25	-0,90	-1,15	-1,16
-2,59	-0,78	-0,79	-1,43	-1,83	-1,85

-1,75	-0,53	-0,54	-0,45	-0,58	-0,58
-1,26	-0,38	-0,39	-0,40	-0,51	-0,51
-0,36	-0,11	-0,11	0,01	0,01	0,01
-0,20	-0,06	-0,06	0,03	0,03	0,03
-1,13	-0,34	-0,35	-0,38	-0,49	-0,50
-1,43	-0,43	-0,44	-0,41	-0,53	-0,53
-0,49	-0,15	-0,15	-0,01	-0,01	-0,01
-3,81	-1,16	-1,20	-0,66	-0,85	-0,89
-1,20	-0,36	-0,37	-0,39	-0,50	-0,50
0,35	0,11	0,11	0,29	0,37	0,38
-0,23	-0,07	-0,07	0,02	0,03	0,03
0,28	0,09	0,09	0,28	0,36	0,37
-0,71	-0,22	-0,22	0,06	0,08	0,08
4,70	1,43	1,44	1,25	1,59	1,61
4,48	1,36	1,37	1,22	1,56	1,57
-0,39	-0,12	-0,12	0,00	0,00	0,00
-4,00	-1,21	-1,22	-0,89	-1,14	-1,14
2,31	0,70	0,71	0,79	1,01	1,02
-0,30	-0,09	-0,09	0,01	0,02	0,02
-5,16	-1,56	-1,58	-1,32	-1,69	-1,70
-1,20	-0,36	-0,37	-0,39	-0,50	-0,50
-1,95	-0,59	-0,60	-0,47	-0,60	-0,62
-1,39	-0,42	-0,43	-0,41	-0,53	-0,53
6,61	2,00	2,02	1,59	2,03	2,05
0,20	0,06	0,06	0,17	0,21	0,21
-1,39	-0,42	-0,43	-0,41	-0,53	-0,53
-4,97	-1,51	-1,51	-1,30	-1,66	-1,67
-4,45	-1,35	-1,37	-0,94	-1,20	-1,22
-2,80	-0,85	-0,86	-0,55	-0,70	-0,71
-4,74	-1,44	-1,45	-1,28	-1,63	-1,64
-1,93	-0,59	-0,59	-0,30	-0,38	-0,38
0,67	0,20	0,21	0,33	0,42	0,42
-1,19	-0,36	-0,36	-0,09	-0,12	-0,12
5,94	1,80	1,81	1,15	1,47	1,48
1,90	0,58	0,59	0,62	0,79	0,81
-1,95	-0,59	-0,60	-0,47	-0,60	-0,62
-3,90	-1,18	-1,19	-0,88	-1,12	-1,13
-0,20	-0,06	-0,06	0,03	0,03	0,03
3,71	1,12	1,14	0,82	1,05	1,06
-3,97	-1,20	-1,21	-0,89	-1,13	-1,14
-0,20	-0,06	-0,06	0,03	0,03	0,03
-3,11	-0,94	-0,97	-1,49	-1,91	-1,96
1,80	0,55	0,55	0,61	0,78	0,79
-1,77	-0,54	-0,54	-0,28	-0,36	-0,36
9,42	2,85	2,92	1,63	2,08	2,13
-6,10	-1,85	-1,86	-2,32	-2,96	-2,98
-0,33	-0,10	-0,10	0,01	0,01	0,01
-2,74	-0,83	-0,84	-0,54	-0,70	-0,70
-3,00	-0,91	-0,91	-0,57	-0,73	-0,74
-3,80	-1,15	-1,16	-0,87	-1,11	-1,12

0,26	0,08	0,08	0,17	0,22	0,22
5,94	1,80	1,81	1,15	1,47	1,48
-4,74	-1,44	-1,45	-1,28	-1,63	-1,64
-3,71	-1,12	-1,13	-0,86	-1,10	-1,11
2,10	0,64	0,64	0,53	0,68	0,69
-2,33	-0,71	-0,71	-1,40	-1,79	-1,81
-2,03	-0,62	-0,62	-0,31	-0,39	-0,40
0,00	0,00	0,00	0,14	0,18	0,19
-0,84	-0,25	-0,25	-0,05	-0,07	-0,07
-2,30	-0,70	-0,70	-1,40	-1,79	-1,81
-1,06	-0,32	-0,32	-0,08	-0,10	-0,10
5,29	1,61	1,62	1,04	1,33	1,35
8,38	2,54	2,57	1,88	2,40	2,43
-2,93	-0,89	-0,89	-0,57	-0,72	-0,73
1,33	0,40	0,41	0,38	0,48	0,49
3,94	1,19	1,20	0,85	1,09	1,09
5,81	1,76	1,77	1,14	1,45	1,46
1,51	0,46	0,46	0,58	0,74	0,75
-2,08	-0,63	-0,65	-0,49	-0,62	-0,64
-0,95	-0,29	-0,29	-0,06	-0,08	-0,08
-0,98	-0,30	-0,30	-0,06	-0,08	-0,08
-4,16	-1,26	-1,27	-0,91	-1,16	-1,17
0,13	0,04	0,04	0,16	0,20	0,20
-0,71	-0,22	-0,22	0,06	0,08	0,08
-4,29	-1,30	-1,31	-0,92	-1,18	-1,19
-0,65	-0,20	-0,20	-0,03	-0,03	-0,03
-1,93	-0,59	-0,59	-0,30	-0,38	-0,38
-1,04	-0,31	-0,32	-0,37	-0,47	-0,48
9,45	2,86	2,93	1,63	2,09	2,13
-2,93	-0,89	-0,89	-0,57	-0,72	-0,73
3,07	0,93	0,93	0,70	0,90	0,90
0,41	0,12	0,13	0,30	0,38	0,39
-2,21	-0,67	-0,69	-0,50	-0,64	-0,67
0,38	0,11	0,12	0,30	0,38	0,38
0,71	0,21	0,22	0,31	0,39	0,40
4,16	1,26	1,27	0,88	1,12	1,13
-1,61	-0,49	-0,49	-0,26	-0,33	-0,34
5,84	1,77	1,78	1,14	1,46	1,47
1,25	0,38	0,38	0,55	0,70	0,71
-1,84	-0,56	-0,56	-0,29	-0,37	-0,37
-2,62	-0,79	-0,80	-1,44	-1,84	-1,85
4,03	1,22	1,23	0,86	1,10	1,11
3,13	0,95	0,96	0,71	0,91	0,91
-1,80	-0,55	-0,55	-0,28	-0,36	-0,36
-2,03	-0,62	-0,62	-0,31	-0,39	-0,40
-4,39	-1,33	-1,35	-0,93	-1,19	-1,21
6,07	1,84	1,85	1,17	1,49	1,50
1,97	0,60	0,60	0,52	0,66	0,67
0,18	0,06	0,06	0,27	0,35	0,35
6,07	1,84	1,85	1,17	1,49	1,50

-4,87	-1,48	-1,48	-1,29	-1,65	-1,66
3,80	1,15	1,17	1,06	1,35	1,37
-0,84	-0,25	-0,25	-0,05	-0,07	-0,07
-0,10	-0,03	-0,03	0,04	0,05	0,05
